# Mass production of lumenogenic human embryoid bodies and functional cardiospheres using in-air-generated microcapsules

**DOI:** 10.1038/s41467-023-42297-0

**Published:** 2023-10-21

**Authors:** Bas van Loo, Simone A. ten Den, Nuno Araújo-Gomes, Vincent de Jong, Rebecca R. Snabel, Maik Schot, José M. Rivera-Arbeláez, Gert Jan C. Veenstra, Robert Passier, Tom Kamperman, Jeroen Leijten

**Affiliations:** 1 University of Twente, TechMed Centre, Department of Developmental BioEngineering, Enschede, The Netherlands; 2 University of Twente, TechMed Centre, Department of Applied Stem Cell Technology, Enschede, The Netherlands; 3 Radboud University, Radboud Institute for Molecular Life Sciences, Faculty of Science, Department of Molecular Developmental Biology, Nijmegen, The Netherlands; 4 University of Twente, TechMed Centre, Max Planck Center for Complex Fluid Dynamics, BIOS Lab-on-a-Chip Group, Enschede, The Netherlands; 5 Leiden University Medical Centre, Department of Anatomy and Embryology, Leiden, Netherlands; 6 IamFluidics B.V., De Veldmaat 17, 7522NM Enschede, The Netherlands

**Keywords:** Tissue engineering, Stem-cell biotechnology, RNA sequencing, Biomedical engineering, Microfluidics

## Abstract

Organoids are engineered 3D miniature tissues that are defined by their organ-like structures, which drive a fundamental understanding of human development. However, current organoid generation methods are associated with low production throughputs and poor control over size and function including due to organoid merging, which limits their clinical and industrial translation. Here, we present a microfluidic platform for the mass production of lumenogenic embryoid bodies and functional cardiospheres. Specifically, we apply triple-jet in-air microfluidics for the ultra-high-throughput generation of hollow, thin-shelled, hydrogel microcapsules that can act as spheroid-forming bioreactors in a cytocompatible, oil-free, surfactant-free, and size-controlled manner. Uniquely, we show that microcapsules generated by in-air microfluidics provide a lumenogenic microenvironment with near 100% efficient cavitation of spheroids. We demonstrate that upon chemical stimulation, human pluripotent stem cell-derived spheroids undergo cardiomyogenic differentiation, effectively resulting in the mass production of homogeneous and functional cardiospheres that are responsive to external electrical stimulation. These findings drive clinical and industrial adaption of stem cell technology in tissue engineering and drug testing.

## Abstract (web summary)

Current methods to generate spheroids are associated with low production throughputs, limiting clinical and industrial translation. Here the authors present a clean ultra-high-throughput in-air microfluidic platform for mass production of lumenogenic embryoid bodies and functional cardiospheres.

## Introduction

Organoids are engineered 3D miniature tissues characterized by tissue or organ structures, which can advance our fundamental understanding of human development and have the potential to revolutionize our healthcare system^1–4^. Specifically, organoids enable the study and treatment of diseases in a biomimetic, human-based, patient-specific, ethical, and animal-free manner^1,5,6^. Organoids have, amongst others, been successfully applied to study infectious and hereditary diseases, screen active pharmaceutical ingredients, model cancer, and develop personalized therapies including regenerative medicine approaches^7–19^. Despite these successes, the translation of organoids to many real-world applications has been hampered by our inability to upscale the engineering of organoids while maintaining reproducibility and quality^20^.

Cell fate within embryoid bodies (EBs) and organoids is highly dependent on morphogenic signaling. Therefore, control over their composition, size, and shape is essential to recapitulate organogenesis in vitro^21^. Yet, engineering well-defined microenvironments that offer size-controlled, reproducible, and scalable EB and organoid production still remains among the biggest hurdles for clinical and industrial translation of this technology^22,23^. Lab-scale EB and organoid production conventionally rely on well-plate-format suspension cultures where stem cells are grown atop low-adhesive (micropatterned) materials^24,25^ or within hydrogels^26^, resulting in limited size control. Compartmentalization in (micro)wells^3,27–31^ or within hanging droplets^32^ can offer improved size control. While these studies frequently claim high throughput, they often rely on batch-based processes that can only facilitate limited and small-scale investigative studies, which are unable to facilitate large-scale studies, clinical translation, and industrial valorization^20^. In contrast, microfluidic production processes allow for continuous production of size-controlled organoids^20^. Consequently, microfluidic encapsulation of (human) pluripotent stem cells (hPSCs) into semi-permeable and chemically defined microcapsules holds the potential for scalable and size-controlled organoid production. As an additional benefit, microencapsulation of pluripotent stem cells in microcapsules also prevents the clumping/fusion of engineered organoids, which currently adversely affects the upscaling of suspension cultures^33^. Currently available microfluidic microencapsulation approaches often claim high throughput while operating in the dripping regime, which is archetyped by limited throughputs in the µl/min range. Furthermore, conventional microfluidic technologies are associated with reliance on emulsifications using oils and surfactants^34–37^ which are known for immune adjuvant effects^36^, and contribute to the development of autoimmune diseases^36^. While microfluidic organoid production approaches are moving toward all-in-water technologies, these often rely on operation in dripping regime and its inherent limited throughputs that are in the µl/min range^38–43^ or have difficulties in achieving monodispersity^44–48^. Due to this combination of low-throughput, low control or a non-clean (e.g., oil and surfactant-containing) production process, these technologies do not support clinically or industrially translation of organoid production. Consequently, an innovative microfluidic technology that allows for scalable, size-controlled, reproducible, and clean (e.g., oil-free and surfactant-free) organoid production has remained urgently wanted.

In this work, we report on the ultra-high-throughput generation of uniform and functional pluripotent stem cell-derived EBs and cardiospheres using a production platform based on in-air microfluidics (IAMF). Specifically, we pioneer the in-air coalescence of three independently tunable liquid microjets to enable the continuous on-the-fly encapsulation of (pluripotent stem) cells in ultrathin-shelled hydrogel microcapsules. Due to its oil-free, chip-free, and jet-based nature, the triple-jet IAMF method overcomes the impermeability^49^, material degradation^49^, biocompatibility^35,36,50–52^, and throughput challenges of current on-chip stem cell compartmentalization approaches with ml/min-range throughputs, while retaining high control and resolution. This unique combination distinguishes the triple-jet in-air microfluidic technology from conventional microfluidics by offering both true scalability and accurate control, which is essential for clinical and industrial translation. The microcapsules offer a highly lumenoconductive microenvironment as near 100% efficient lumen formation (i.e., lumenogenesis) and facilitate radial cell/nucleus polarization under physically and chemically defined conditions. Moreover, upon chemical stimulation, the cell spheroids undergo cardiomyogenic differentiation effectively yielding large amounts of functional (i.e., beating) cardiospheres that are responsive to external electrical stimulation. In short, we report on the continuous, ultra-high-throughput production of homogeneous, size-controlled, cellular aggregates, cavitated spheroids, and functional cardiospheres, which is anticipated to drive the clinical and industrial adaption of spheroid and organoid technology in application areas that need ultra-high-throughput tissue production such as tissue engineering, drug testing, and cultured meat.

## Results and discussion

### Triple-jet IAMF enables high-throughput fabrication of monodisperse, hollow, thin-shelled microcapsules for controlled spheroid, embryoid body, and cardiosphere formation

**Fig. 1:**
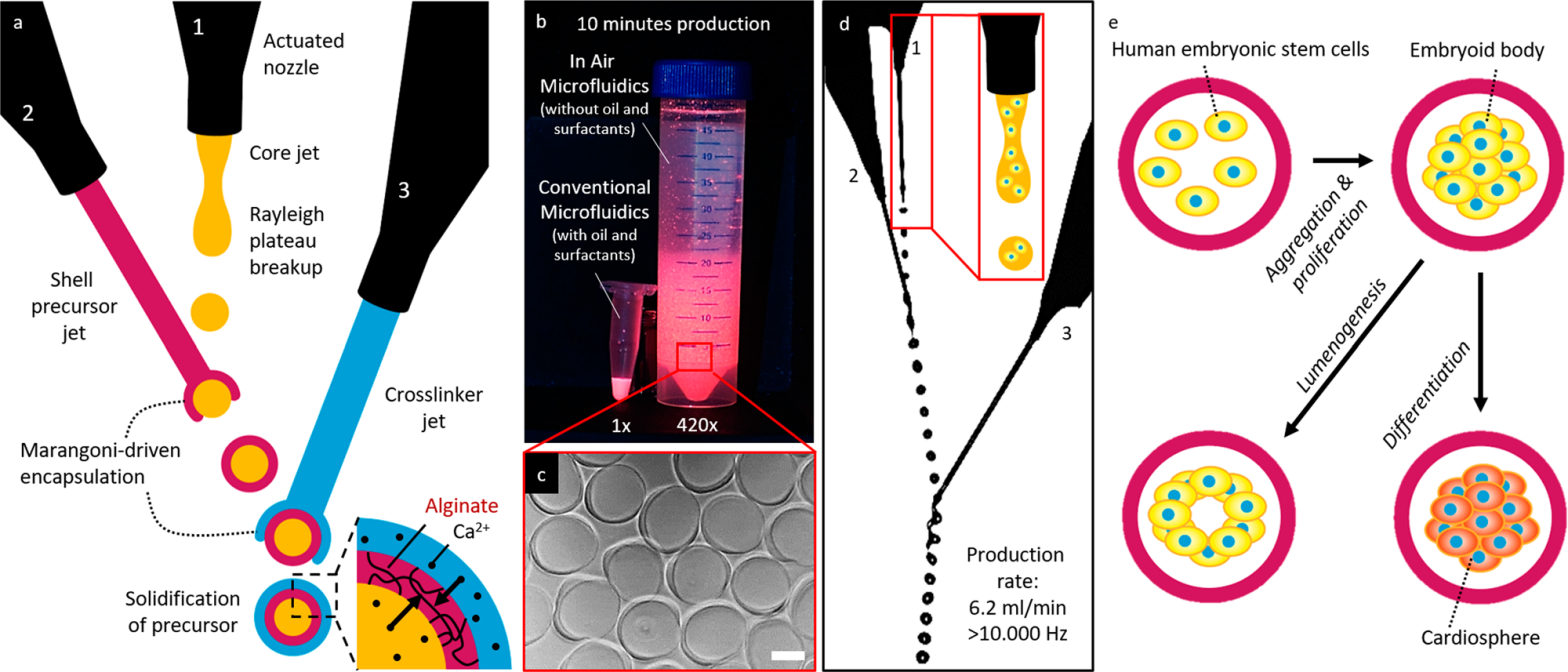
Triple-jet in-air microfluidic production of cellular spheroid-forming hollow microcapsules. **a** Schematic of three nozzle in-air microfluidics for the production of alginate microcapsules. **b** Photograph of fluorescently labeled microcapsules that could be produced within a 10-min time span using either in-air microfluidics or conventional microfluidics^117^. **c** Microphotograph of hollow microcapsules produced using In-Air Microfluidics. **d** Microphotograph of in-air production of triple-layered aqueous droplets. **e** By addition of human pluripotent stem cells to the core microjet cell-laden microcapsules are formed, which self-assemble into cellular spheroids that autonomously self-organize into cavitated embryoid bodies and can be differentiated into functional cardiospheres. Scale bar is 200 µm.

In-air microfluidics (IAMF) is a recently invented jet-based microfluidic technology that enables ultra-high-throughput, oil-free, chip-free, and cytocompatible production of monodisperse solid microparticles^53^. However, creating monodisperse, hollow, micrometer-sized hydrogel capsules (e.g., microcapsules) has remained a challenge and has thus prevented its use in organoid production. Conventionally, in IAMF, a first monodisperse hydrogel precursor droplet train is produced by superimposing a vibration on a jetting micronozzle that is combined with a second crosslinker jet, which results in a stream of monodisperse compound droplets. The use of a rapidly crosslinking material combination (e.g., alginate and a divalent cation such as calcium) results in on-the-fly stabilized particles of controlled size. However, producing microcapsules with the existing IAMF approaches cannot be reliably achieved as they are associated with the merging of capsules into larger, poorly defined multi-core constructs (Supplementary Fig. S1)^53^. Here, we report on an IAMF strategy that prevents the merging of microcapsules via the introduction of a third microjet, which allows for the stable in-air production of triple-layered aqueous droplets (Fig. 1a, Supplementary Fig. S2 Supplementary Video 1). The addition of a third microjet enables the Marangoni-driven encapsulation of the droplets to occur twice, which enabled simultaneous inside-out and outside-in crosslinking of the alginate shell (i.e., middle phase) by calcium ions in the crosslinker liquids (i.e., inner and outer phases). Importantly, this allows for the jet-based production of thin-shelled microcapsules at a rate that is more than two orders of magnitude faster than competing fabrication techniques (e.g., conventional microfluidics^54^) (Fig. 1b, c).

To demonstrate that in-air-produced hollow microcapsules can function as cellular spheroid-forming bioreactors, human pluripotent stem cells (hPSCs) were introduced into the first microjet (Fig. 1d, e). After successful encapsulation of hPSCs within calcium-alginate microcapsules at a production frequency of over 10 kHz, the cells rapidly aggregated and subsequently self-organized to form a lumen (Fig. 1e)^55^. Furthermore, encapsulated hPSCs could be differentiated into functional cardiospheres that contain highly organized sarcomeres.

### Achieving ultrafast, controlled, and clean microcapsule production by balancing Marangoni flows and ionic crosslinking in flying droplets

**Fig. 2:**
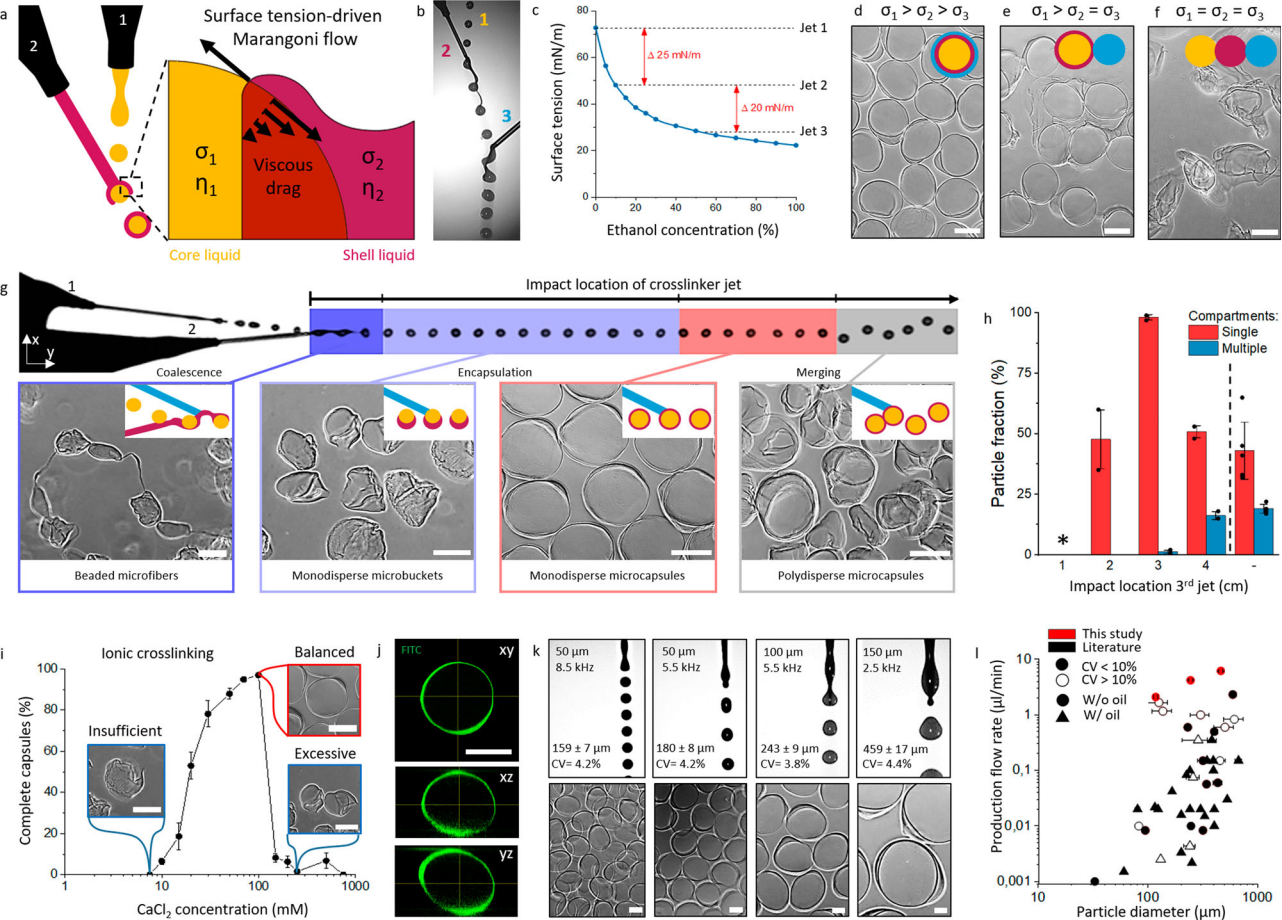
Ultra-high-throughput production of hollow microcapsules using in-air microfluidics. **a** Schematic of the surface tension-driven in-air encapsulation process. **b** Microphotograph of in-air aqueous layering process that enables microcapsule production. **c** Used surface tension differences between microjets were obtained using distinct EtOH concentrations. **d**–**f** Microphotographs of microparticles formed with stepwise tuning of microjet surface tensions. Schematics indicate the surface tension-driven encapsulation status per condition. Yellow indicates the core solution, red indicates the shell precursor solution and blue indicates the crosslinker solution. **g** Schematic showing the effect of the impact location of the crosslinker microjet with microphotographs of resulting micromaterials. Insert schematics indicate the surface tension-driven encapsulation status per condition. Yellow indicates the core solution, red indicates the shell precursor solution and blue indicates the crosslinker solution. **h** Quantification of microcapsule formation efficiency and number of compartments per microcapsule dependent on third jet impact location (*n* = 60 microparticles per condition). * Indicates the formation of beaded microfibers void of individual microcapsules. Data is presented as mean values ± SD. **i** Analysis and microphotographs of insufficient, balanced, and excessive ionic crosslinking at different core-microjet CaCl_2_ concentrations (*n* = 350 microparticles). Data is presented as mean values ± SD. **j** Confocal analysis of a representative FITC-conjugated dextran (2000 kDa)-alginate microcapsule (*n* = 3 experiments). A representative microparticle **k** Microphotographs and analysis of droplet formation and resulting microcapsules using different nozzle sizes and different piezoelectric actuator frequencies (*n* > 100). **l** Literature study for continuously produced hollow core-shell microcapsules. Data from this study is indicated in red, while data from literature is indicated in black. CV < 10% Is indicated in filled data points, while CV > 10% is indicated in empty data points. Horizontal bars indicate the particle diameter range found in literature. Circular data points indicate studies without the use of oil, while triangular data points indicate studies with the use of oil. Scale bars represent 100 µm.

In-air encapsulation of liquid droplets by another liquid phase can be achieved via Marangoni spreading, which drives low-surface tension liquids to encapsulate high-surface tension liquids (Fig. 2a). We hypothesized that consecutive spreading of alginate and calcium-containing solutions over a calcium-laden droplet could be achieved by exploiting two distinct surface tension gradients, as Marangoni spreading is proportional to the difference in surface tension (*σ)*^56^. Indeed, on-the-fly colliding water droplets containing 50 mM calcium chloride and 10% dextran (surface tension *σ*_*1*_ ~ 73 mN/m) with a liquid jet containing 0.2% (w/v) sodium alginate and 10% ethanol (*σ*_*2*_ ~ 48 mN/m), which subsequently collides with a liquid jet containing 100 mM calcium chloride and 55% ethanol (*σ*_*3*_ ~ 28 mN/m) effectively yielded triple-layered aqueous droplets that ionically crosslinked into water-filled thin-walled calcium-alginate microcapsules within milliseconds (Fig. 2b–d). Creating two surface tension gradients was indeed key to producing individual monodisperse capsules, as confirmed by the merging of capsules when using *σ*_*1*_ > *σ*_*2*_ = *σ*_*3*_ (Fig. 2e) and the absence of capsules when using *σ*_*1*_ = *σ*_*2*_ = *σ*_*3*_ (Fig. 2f, Supplementary Fig. S2).

Systematic examination of the on-the-fly microcapsule formation by triple-jet IAMF revealed that the relative impact location of the third (crosslinker) jet had a dominant effect on microparticle morphology (Fig. 2g and Supplementary Fig. S3). Quantification reveals that 99.5 ± 0.5% of the microcapsules contained a single compartment upon third-jet optimization, which is in stark contrast to the 43 ± 12% obtained using a two-jet approach (Fig. 2h). In principle, the third crosslinker jet arrested the in-flight droplet formation and encapsulation processes in its temporally defined state. Specifically, introducing the third jet before full droplet pinch-off occurred resulted in beaded microfibers, whereas coalescing the third jet with the pinched-off calcium-alginate droplets resulted in partly formed (i.e., microbuckets) or fully formed microcapsules. Coalescing the third jet too far downstream produced polydisperse multi-core capsules that looked similar to microcapsules produced with conventional two-jet IAMF. To further characterize and optimize the in-air production of ionically crosslinked microcapsules, the concentration of calcium in the core liquid of the triple-layered aqueous droplets was varied. A calcium concentration of <10 mM associated with the collapse of the core upon impact with the third jet or with the collection bath (Fig. 2i). A calcium concentration of >100 mM impaired the formation of microcapsules, which can be attributed to the accelerated calcium-alginate crosslinking when using higher calcium concentrations causing the viscosity of the alginate solution to increase, thereby causing larger viscous drag and hampering Marangoni spreading^57^. This resulted in the reproducible production of crescent-shaped microbuckets. Indeed, reducing the calcium diffusion speed by increasing viscosity through adding dextran to the core aided the stable formation of intact calcium-alginate microcapsules (Supplementary Fig. S4). Of note, adding a viscous crowder (e.g., 10% dextran) to the core microjet also reduced droplet deformations upon pinch-off owing to increased liquid viscosity of the triple-layered droplet’s core (Supplementary Fig. S4). Overall, these results revealed that balancing of liquid viscosity, surface tension, crosslinking rate, and collision timing enable optimized ultra-high-throughput production of hollow microcapsules.

Single-core microcapsules composed of FITC-labeled alginate revealed the micrometer thin (6.4 ± 3.9 µm) nature of the microcapsule’s hydrogel shell, which was visualized using confocal microscopy (Fig. 2j, Supplementary Fig. S5A). Microcapsule size was monodisperse (coefficient of variation; CV < 5%) and could be produced over a wide range of diameters, for example, 159 ± 7 µm, to 243 ± 9 µm, and to 459 ± 17 µm when using 50 µm, 100 µm, and 150 µm nozzles for the core liquid, respectively (Fig. 2k and Supplementary Fig. S5B). Furthermore, capsule size could be fine-tuned by adjusting the nozzle vibration frequency. Interestingly, adjusting the frequency also provided control over the number of hollow compartments per microgel. While a typical hollow microgel containing a single compartment was achieved with a stable droplet train using a frequency of 5.5 kHz, other frequency regimes produced droplet trains where droplets traveled in doublets (Supplementary Fig. S6), or even triplets. This results in 83 ± 2% dual-core microcapsules at 2.7 kHz (Supplementary Fig. S6), and 88 ± 6% triple-core microcapsules at 2 kHz (Supplementary Fig. S6). Consequently, triple-jet IAMF allowed for ultra-high-throughput production of multi-compartment microcapsules, which offers opportunities for various biomedical, chemical, pharmaceutical, and cosmetic applications^58–61^. Of note, triple-jet IAMF technology also facilitates such variety of uses as it can produce compartmentalized micromaterials from materials other than alginate, as demonstrated by the fabrication of microcapsules composed of interpenetrating hydrogel networks of ionically crosslinked calcium-alginate and photocrosslinked poly(ethylene glycol) (PEGDA) or calcium-alginate and enzymatically crosslinked dextran-tyramine (Dex-TA) (Supplementary Fig. S7).

To determine the operational window for monodisperse hollow microcapsule production, flow rates were systematically investigated and optimized. Single compartment hollow microcapsules of 180 ± 8 µm having a highly circular morphology (circularity > 0.9) and monodisperse size (CV < 5%) were obtained at core and shell microjet flow rates of 900 µm/min and 1100 µl/min, respectively (Supplementary Fig. S8). Importantly, an extensive literature study^39–49,54,62–85^ revealed and confirmed that no other platform rivals triple-jet IAMF microcapsule production as in terms of production throughput, resolution, oil-free production, and monodispersity (Fig. 2l, Supplementary Fig. S9). Specifically, triple-jet IAMF represents the first platform that offers ultra-high-throughput production (2 ml/min, 4.2 ml/min and 6.4 ml/min for 159 µm, 243 µm and 459 µm microcapsules, respectively), high resolution (i.e., the possibility for producing particles <500 µm) and highly monodisperse (CV < 5%) microcapsules in an oil-free manner.

### Engineered microcapsules as bioreactors for mass production of long-term stable cellular spheroids

**Fig. 3:**
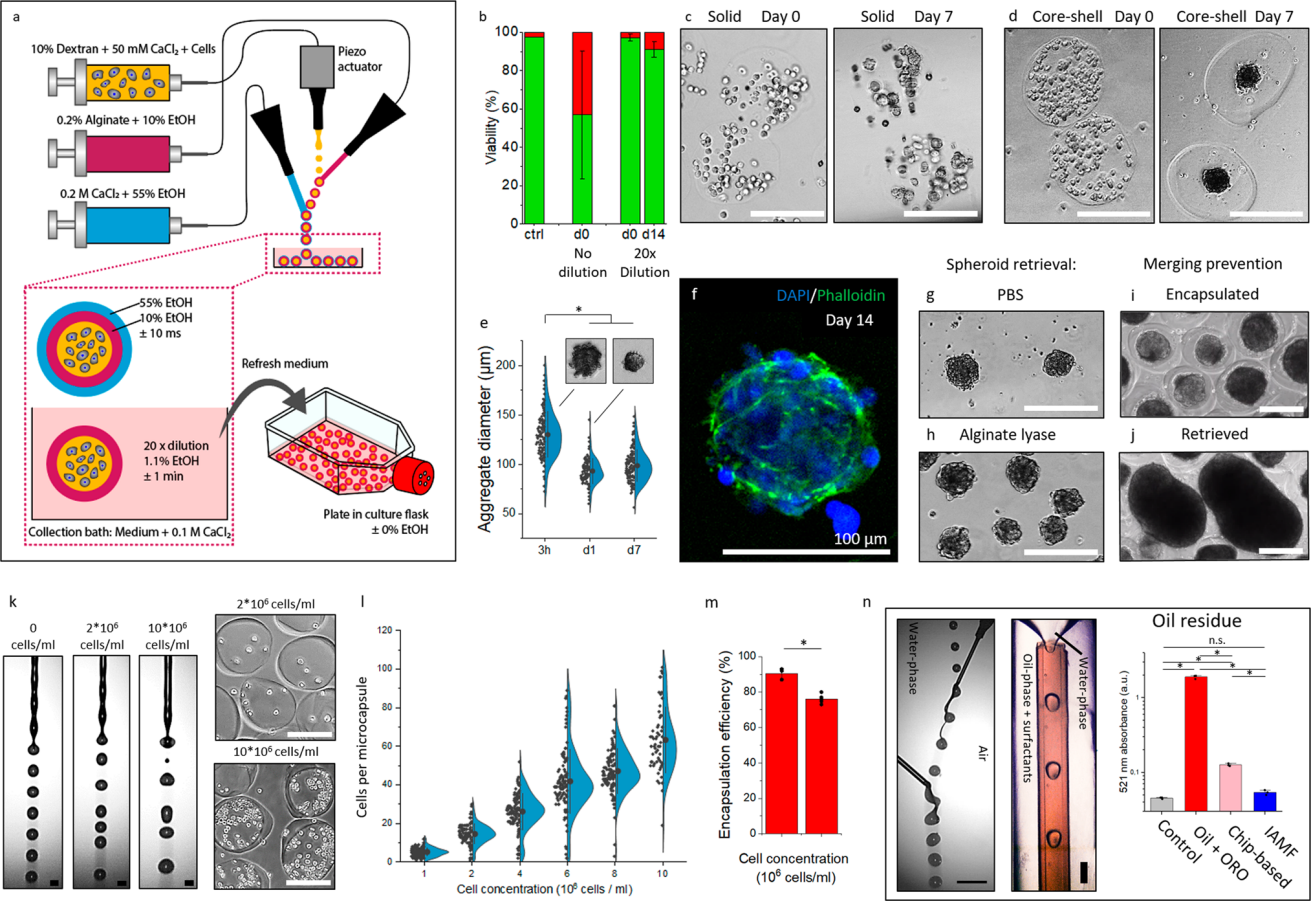
Clean mass production of viable spheroids using in-air-generated cell-laden microcapsules. **a** Schematic of triple-jet in-air microfluidic cell encapsulation in microcapsules. **b** Viability quantification of encapsulated 3T3 fibroblasts with and without 20x dilution upon collection (*n* = 85 microcapsules). Data is presented as mean values ± SD. Microphotographs of **c** solid microgels and **d** hollow microcapsules containing 10^7^ 3T3 fibroblasts/ml after 0 and 7 days culture. **e** Quantification and microphotographs of 3T3 fibroblast spheroid diameter within microcapsules during 7 days of culture (*n* = 60, *p* = 1.37E-29). Data is presented as mean values ± SD. **f** Confocal fluorescence microphotograph of a microcapsule and its 3T3 spheroid of which the nuclei are stained with DAPI (blue) and its F-actin is stained with phalloidin (green) after 14 days of culture. Microphotographs of spheroids of which their respective microcapsule was instantly dissolved using **g** PBS or **h** alginate lyase after 14 days of culture. Microphotographs of **i** non-merging encapsulated spheroids and **j** merging de-encapsulated spheroids. **k** Microphotographs of droplet formation using solutions containing 0, 2*10^6^ and 10^7^ cells/ml. **l** Quantification of cells per microcapsule for various cell concentrations ranging up to 10^7^ cells/ml (*n* = 60). Data is presented as mean values ± SD. **m** Encapsulation efficiency of microcapsule production using 2*10^6^ or 10^7^ cells/ml was quantified (*n* = 60, *p* = 1.01E-4). Data is presented as mean values ± SD. **n** Microphotograph of conventional oil-based microfluidic droplet formation using oil and surfactants, microphotograph of samples obtained with oil-based microfluidics using Oil Red O (ORO) stained oil, and absorbance at 521 nm for residue oil quantification. The chip-based sample was obtained through three thorough washing steps (*n* = 3). Data is presented as mean values ± SD. Significance was determined based on one-way ANOVA analysis. The significance of *p* < 0.05 is indicated by *. A.U. Indicates arbitrary units. Scale bar is 200 µm unless stated otherwise.

Triple-jet IAMF was then explored for the encapsulation of living cells. Specifically, a well-characterized fibroblast cell line (3T3) was used to establish the cytocompatibility of the microcapsule production process. On-the-fly encapsulated cells were collected in a bath composed of cell culture medium to ensure near-instant >20 times dilution of ethanol levels, which reached maximally ~1% concentrations. While a 1% ethanol concentration is considered non-toxic when exposing it to cells shorter than 24 h, microencapsulated cells were washed extensively within 1 min upon collection to further ensure reproducible cytocompatibility (Fig. 3a)^86–88^. Similarly, physiological calcium concentrations were retained upon the before-mentioned extensive washes within 1 min of collection. Indeed, triple-jet IAMF offered excellent cytocompatibility with the viable cell fraction as high as 97 ± 2% directly post-encapsulation and 91 ± 4% after 14 days of culturing (Fig. 3b). Permeability experiments with Dextran-FITC conjugates revealed excellent permeability with an approximate diffusion cutoff of 2000 kDa (Supplementary Fig. S10), While conventional matrix-type (i.e., solid) calcium-alginate microgels did not support spheroid formation of 3T3 cells (Fig. 3c), hollow microcapsules readily enabled rapid cell aggregation, which resulted in 3D microtissues formation with aggregate diameters of 99 ± 16 µm (CV = 16%) (Fig. 3d–f). The cell spheroids could be straightforwardly retrieved from the microcapsules in an enzyme-free manner by exposing the microcapsules to phosphate-buffered saline (PBS) solution, which dissolved the calcium-alginate by competitively binding and extracting the calcium that ionically crosslinked the polymer network to form calcium phosphate, which is not dissolvable in water (Fig. 3g). In addition, cell spheroids could be retrieved by bio-orthogonal enzymatic digestion of the alginate using alginate lyase (Fig. 3h). Notably, while encapsulated spheroids remained separate (Fig. 3i), retrieved non-encapsulated spheroids merged upon further culture (Fig. 3j), which underlined the advantage of microcapsule culture to obtain and maintain controlled spheroid sizes^20^. The optimized microencapsulation platform was then tested for different cell concentrations. Although introducing cells into the polymer solution associates with introducing a granularity into the liquids that at high concentrations could adversely affect jet breakup (Fig. 3k), 3T3 fibroblasts using cell concentrations ranging from 10^6^ cells/ml to 10^7^ cells/ml, which corresponded to payloads of 5 ± 2 to 63 ± 17 cells/microcapsule, readily allowed for reproducible and stable microencapsulation (Fig. 3l, m and Supplementary Fig. S11).

A key advantage of IAMF over conventional chip-based microfluidics encapsulation strategies is its oil-free nature. As oil residue is known to comprise cell viability^35,37^, associate with immune adjuvant effects^36^, and contribute to the development of autoimmune diseases^36^, the clinical translational potential of conventional microfluidic strategies is limited. We demonstrated that micromaterials produced with IAMF-based cell encapsulation do not contain oil while microcapsules produced via chip-based microfluidic emulsification, even after extensive washing (i.e., >10^6^ times diluted via three washing steps using >100x excess of liquid) contains oil residues (Fig. 3n). Together, these results highlight the clean nature of triple-jet IAMF encapsulation, which is expected to facilitate clinical and industrial translation of this technology for the clean mass production of 3D microtissues for clinical or biomedical applications.

### Engineering embryoid bodies using size-controlling microcapsules

**Fig. 4:**
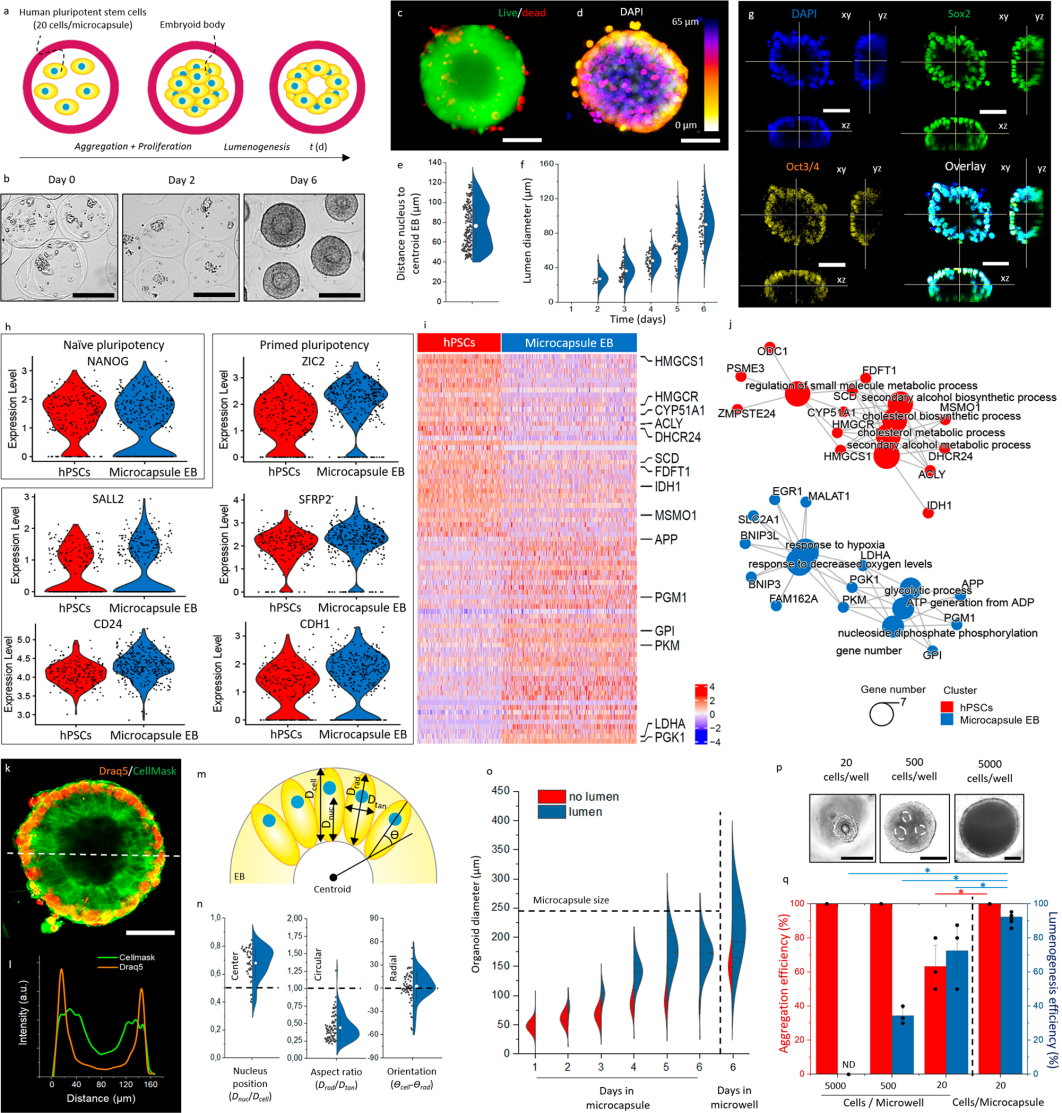
Microencapsulated human pluripotent stem cells self-organize into polarized cavitated embryoid bodies. **a** Schematic and **b** representative microphotographs of aggregation, compaction, and cavitation of human pluripotent stem cell (hPSC)-laden microcapsules. (*n* = 3 experiments) **c** Fluorescence microphotograph of embryoid body (EB) stained with calcein AM (green) and ethidium homodimer (red) to visualize life and dead cells, respectively. **d** Confocal fluorescence Z-projection of a DAPI-stained cavitated EB. **e** Quantification of distance between nuclei and centroid of EBs (*n* = 250 cells). Data presented as mean values ± SD. **f** Quantification of the diameter of the lumen of microencapsulated EBs over time (day 2: *n* = 8, other conditions: *n* = 48). Data presented as mean values ± SD. **g** Confocal fluorescence microphotographs of EB fluorescently stained for nuclei (blue), SOX2 (green), and Oct3/4 (yellow). **h** Gene expression analysis for naïve pluripotency (NANOG) and primed pluripotency (SALL2, CD24, ZLC2, SFRP2, CDH1) for conventional microwell cultured EB control (red) and microencapsulated EBs (blue) (hPSCs *n* = 208, Microcapsule EB *n* = 333). **i** Single-cell RNA sequencing heatmap of marker genes with highlighted genes related to biosynthesis (HMGCS1-MSMO1) and glycolysis (APP-PGK1). **j** GO-term networks of differences between conventional microwell cultured EB control (red) and microencapsulated EBs (blue). **k** Fluorescence confocal microphotograph of EB stained with Draq5 (orange) for nuclei, and Cellmask (green) for cell membrane. White dotted line indicates **l** histogram of Draq5 and Cellmask. A.U. Indicates arbitrary units. **m** Schematic of EB with distance of nucleus perpendicular to the cavity (d_Nucleus_), length of the cell perpendicular to the cavity (d_Cell_), distance of the cell radial (d_radial_) and perpendicular (d_perpendicular_), and orientation of the cell compared to the EB’s centroid (ϴ_cell_-ϴ_rad_). **n** Quantification of nucleus position and the aspect ratio and orientation of the cells. (*n* = 57 cells) Data is presented as mean values ± SD. **o** Quantification of embryoid body diameter over time, with individual denomination for EBs without cavity (red) and with cavity (blue) (*n* > 110). **p** Microphotographs hPSCs aggregates at different cell concentrations formed using the conventional microwell technique. **q** Quantification of aggregation and cavitation process of hPSCs at different cell concentrations using microwells and in-air microencapsulation (microwells: *n* = 30 microtissues, microcapsules: *n* = 175 microtissues). Data presented as mean values ± SD. Black scale bar represents 100 µm. White scale bar represents 50 µm.

We then investigated whether microcapsules could act as bioreactors, enabling mass production of high-quality EBs. To this end, microcapsules were laden with ~20 human pluripotent stem cells and suspension-cultured in Essential 8 (E8) stem cell medium, which was supplemented with PVA and ROCK inhibitor during the first 24 h only (Fig. 4a). Microencapsulated hPSCs aggregated into compact 3D cell spheroids within 2 days (Fig. 4b, Supplementary Fig. S12). Within 6 days, the vast majority (92 ± 1%) of hPSC spheroids underwent lumenogenesis, which was progressively developing and did not associate with the formation of a necrotic core (Fig. 4c–f). Specifically, the volume of the average lumen grew by >30-fold between day two and six of the microencapsulated suspension culture (Fig. 4f). In mammalian embryogenesis, lumenogenesis of the blastocyst is essential for the formation of the trophoblast lineage, its separation from the inner cell mass, and the subsequent formation of the three germ layers of the embryo proper^89^. Subsequently, cavitation plays a key role at the rosette stage in the formation of the amniotic cavity^90^. Hence, high-efficiency lumenogenesis is of high value for both embryonic self-organization and EB-mediated organoid formation^25,27,28,91–94^. The pluripotent character of the hPSC spheroids was confirmed by pluripotency markers Sox2 and Oct3/4, which were expressed in 98% ± 4% and 89% ± 9% of the PSC-microspheres, respectively, which was comparable to pluripotency marker levels observed in conventional monolayer cultures (100% ± 1% Sox2 and 94% ± 10% Oct3/4) (Fig. 4g and Supplementary Fig. S13).

hPSC-Spheroids cell population variation was investigated through single-cell RNA sequencing, which revealed that the populations of single cells of microencapsulated hPSCs and conventional hPSCs were near-identical regarding the transcriptional expression of naïve pluripotency markers (e.g., *NANOG*) and primed pluripotency (e.g., *SALL2*, *CD24*, *ZLC3*, *SFRP2*, and *CDH1*) (Fig. 4h). This is advantageous as it indicates that this production platform is highly comparable in quality to conventional techniques while supporting upscaling via suspension culture. Although highly similar, several interesting distinctions were observed: while gene expression of biosynthesis markers (*HMGCS1*, *HMGCR*, *CYP51A1*, *ACLY*, *DHCR24*, *SCD*, *FDFT1*, *IDH1*, and *MSMO1*) was higher in conventional non-encapsulated hPSCs, the gene expression of glycolytic markers (*APP*, *PGM1*, *GPI*, *PKM*, *LDHA*, and *PGK1*) was higher in encapsulated EBs (Fig. 4i, j). This is likely of value as high glycolytic flux is critical in obtaining and maintaining pluripotency^95–97^, and it is known that primed pluripotent stem cells are typically exclusively glycolytic as compared to naïve pluripotent stem cells, which rely relatively more on oxidative metabolism^98,99^.

To further investigate the cytoarchitecture of the self-organizing spheroids, we visualized and measured the nucleus position, cell orientation, and cell aspect ratio (Fig. 4k–m, Supplementary Fig. S13). Cells within spheroids were consistently characterized by peripherally located nuclei with a *d*_*nucleus*_/*d*_*cell*_ of 0.68 ± 0.11, an aspect ratio *d*_*radial*_/*d*_*tangent*_ of 0.44 ± 0.18 and a predominantly radial orientation ϴ_cell_-ϴ_rad_ of 2.3° ± 19.7° (Fig. 4l–n). This highly anisotropic cytoarchitecture indicated the formation of columnar epithelium, which, combined with the observed lumenogenesis and positive primed pluripotency markers including *OTX2*, *FGF2*, *ZIC2* and *SFRP2* (Fig. 4h)^89^, indicate resemblance to rosette-stage epiblast^90^. However, more research is needed to confirm this hypothesis.

Timelapse observations of growing microencapsulated hPSCs revealed that lumenogenesis was strongly correlated with a physical size threshold. Specifically, lumenogenesis exclusively and rapidly occurred in spheroids with *D*_*spheroid*_ ≳100 µm (Fig. 4o). Spheroid formation and lumen formation were more controlled, homogeneous, and monodisperse in microcapsules as compared to microwell culture, with 172 ± 31 µm (CV 18%) and 92.4 ± 0.8% of lumenogenesis in microcapsules and 198 ± 65 µm (CV 33%) and 72.5 ± 16% of lumenogenesis in microwells. It was observed that when microcapsules became fully confluent (e.g., after 5 days of culture) the size of the cell mass stabilized. Since microcapsule size remained constant upon confluency, this offers an elegant manner of spheroid size control by imposing a designable spatial confinement defined by microcapsule size. To investigate if lumen formation indeed correlated with cell mass size, we prepared small, medium, and large spheroids by seeding 20, 500, and 5000 hPSCs per conical non-cell-adhesive culture chamber. As hypothesized, lumenogenesis efficiency correlated with the number of cells per microwell and thus size (Fig. 4p, q). Specifically, no cavitation was observed in the larger spheroids while 34.4 ± 4.1% of medium-sized encapsulated hPSC spheroids underwent lumenogenesis, albeit in an uncontrolled manner characterized by the formation of multiple lumens of various sizes (Fig. 4p). In marked contrast, only small spheroids developed into single-lumen 3D cell spheroids in a fairly reproducible manner (i.e., 72 ± 16% lumenogenesis efficiency), albeit with significantly lower efficiency than triple-jet IAMF microencapsulated EBs (i.e., 92.4 ± 0.8% lumenogenesis efficiency) (Fig. 4q). This improved performance of 3D microcapsules could potentially be explained by the conformal encapsulation, which unlike microwells controls molecule transport via diffusion by locally preventing convection, therefore allowing for the more efficient buildup of an autocrine microenvironment^100^.

### Engineering monodisperse functional cardiospheres using IAMF-generated bioreactors

**Fig. 5:**
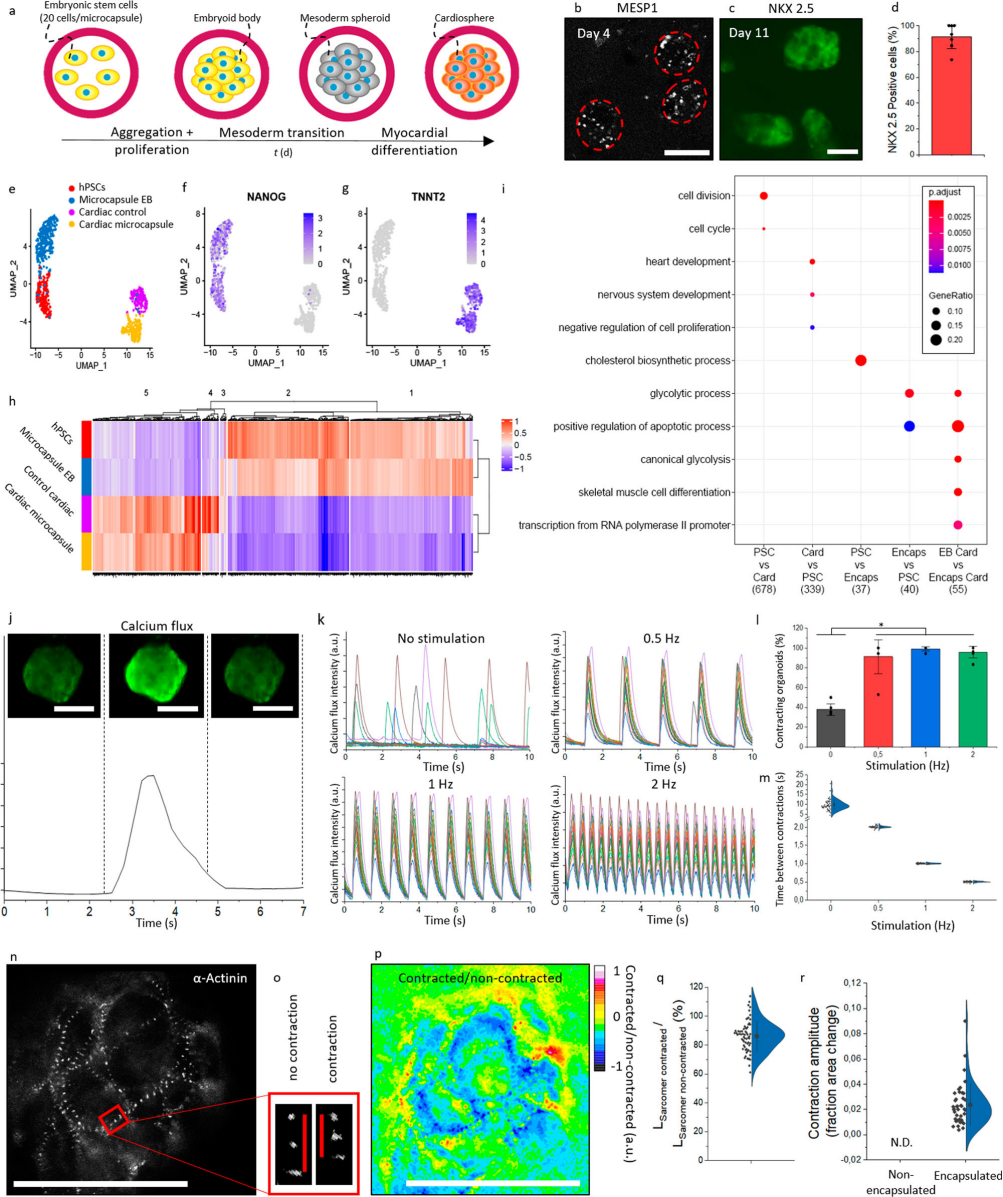
Mass production of functional cardiospheres using in-air-generated human pluripotent stem cell-laden microcapsules. **a** Schematic of aggregation, mesoderm transition, and myocardial differentiation of human pluripotent stem cells (hPSC)-laden microcapsules. **b** Fluorescence microphotograph of MESP1 mesoderm marker in hPSC-laden microcapsules after 4 days of culture. **c** Fluorescence microphotograph of NKX-2.5 myocardial marker in hPSC-laden microcapsules after 11 days of culture. **d** Quantification of NKX-2.5 positive cells after 22 days of encapsulated culture (*n* = 126). Data presented as mean values ± SD. **e** Uniform manifold approximation and projection (UMAP) plot of non-differentiated hPSCs (blue) (*n* = 208), microencapsulated EBs (purple) (*n* = 333), control cardiac culture (red) (*n* = 162) and microencapsulated cardiospheres (green) (*n* = 193). UMAP heatmaps of **f** NANOG and **g** TNNT2. **h** Single-cell RNA sequencing heatmap of marker genes corresponding to identified clusters. **i** Gene ontology heatmap of differential genes per cluster. Columns from left to right: (1) Upregulated in PSC conditions vs cardiac conditions, (2) Upregulated in cardiac conditions vs PSC, (3) Upregulated in hPSC versus pluripotent EB microcapsule, (4) Upregulated in pluripotent EB microcapsule versus hPSC, (5) Upregulated in cardiac EB versus cardiac microcapsule. GO-terms with a gene ratio >0.05 were visualized. *P*-adjust corrected using Benjamini–Hochberg correction for multiple comparisons. **j** Fluorescence microphotographs and analysis of calcium flux by Fluo-4 staining (green). A.U. Indicates arbitrary units. **k** Analysis of calcium flux intensity in encapsulated cardiospheres without stimulation, or with stimulation using an electrical current with a frequency of 0.5, 1, and 2 Hz (*n* = 17). **l** Quantification of contracting encapsulated cardiospheres with or without electrical stimulation (*n* = 120, *p* = 5.19E-6). Data presented as mean values ± SD. **m** Quantification of time between contractions with or without electrical stimulation (*n* = 50). Data presented as mean values ± SD. **n** Fluorescence microphotograph of hPSC α-actinin reporter (mRubyll) in differentiated encapsulated cardiomyocytes after 18 days of culture. **o** Fluorescence microphotograph showing shortening of sarcomeres during contraction in encapsulated hPSC-derived cardiomyocytes. **p** Ratiometric image of contracted/non-contracted microphotographs of encapsulated cardiospheres. **q** Quantification of contraction by assessment of single sarcomeres in encapsulated cardiospheres (*n* = 66 sarcomeres). Data presented as mean values ± SD. **r** Quantification of contraction by assessment of fraction area change of non-encapsulated and encapsulated cardiospheres (*n* = 40 cardiospheres). Data presented as mean values ± SD. Scale bar represents 100 µm.

To further explore the translational potential of IAMF-generated microcapsules as bioreactors, microencapsulated EBs were differentiated into functional cardiospheres. Immediately following aggregation and compaction of on average 20 hPSCs per microcapsules, mesoderm transition and cardiac differentiation were induced by exposing the resulting microencapsulated EBs to mesoderm differentiation medium for 3 days and subsequently to cardiac differentiation medium for 22 days (Fig. 5a). Successful cardiosphere development was investigated and confirmed by visualizing the expression of Mesoderm Posterior BHLH Transcription Factor 1 (MESP1) and homeobox protein NKX2-5 using genetically engineered reporter hPSC cell lines (Fig. 5b, c)^101^. After 22 days, 94.3 ± 0.5% of encapsulated cells expressed Green Fluorescent Protein (GFP) (Fig. 5d), which indicated activation of *NKX2-5* expression and highly efficient cardiac differentiation comparable to previously reported optimized yet low-throughput cardiac differentiation strategies^102^.

Next, the quality of cardiac differentiation within the microcapsules in terms of population heterogeneity was investigated. To this end, single-cell RNA sequencing was performed on cardiac differentiated hPSCs that were cultured in microcapsules or with conventional cardiac culture in cell culture wells, which were compared to undifferentiated hPSCs and microencapsulated cavitated hPSCs (Fig. 5e). It is important to note that conventional cardiac culture in 2D cell culture wells will form 3D tissues and therefore does not resemble traditional monolayer culture. Differentiation toward the cardiac lineage associated with loss of pluripotency markers (e.g., *NANOG*) (Fig. 5f) and transcription of cardiac markers (e.g., *TNNT2*, *RYR2*, and *MYL7)* (Fig. 5g, Supplementary Fig. S14) in both microencapsulated and conventional cardiac culture. Importantly, no notable subpopulation was observed among the microencapsulated cardiospheres, indicating that the hPSC-derived cardiomyocyte population was highly similar at the single-cell level. This is a highly desirable feat as it demonstrates that IAMF-generated microcapsules can act as bioreactors for the mass production of high-quality (e.g., homogeneous) hPSC-derived cardiomyocyte populations.

Heatmap analysis of the genes that were differentially expressed between the obtained single-cell RNA sequencing data sets (i.e., undifferentiated hPSCs, hPSC-derived cavitated EBs, conventional cardiac culture, and microcapsule cardiospheres) revealed that the gene expression profiles of conventional cardiac cultures and microcapsule-mediated mass-produced cardiospheres were near-identical (Fig. 5h, Supplementary Fig. S15). Gene ontology analysis of identified cell clusters revealed that non-differentiated hPSCs differentially expressed genes relating to cell proliferation and metabolism as compared to differentiated cardiospheres (Fig. 5i, Supplementary Fig. S15). Compared to non-differentiated hPSCs, both encapsulated cardiospheres and conventional cardiac culture showed increased expression for cardiac tissue formation markers with no major differences between microencapsulated and conventional conditions. Consequently, this suggested that in-air mass production using bioreactors offers similar high-quality cardiomyocytes and cardiac culture as conventional non-scalable production methods.

Time-resolved microscopy confirmed that microencapsulated cardiospheres underwent spontaneous contraction (Supplementary Video 2), which coincided with a calcium flux as visualized using a fluorescently labeled calcium indicator Fluo-4 AM (Supplementary Video 3 and Fig. 5j). Furthermore, exposure to cyclical electrical stimulation at various physiologically relevant frequencies (0.5, 1 and 2 Hz) resulted in synchronized corresponding contractile behavior of all observed mass-produced cardiospheres at near 100% efficiency (Fig. 5k–m), which corroborated successful cardiac differentiation and function^103^. Interestingly, the possibility of pacing cardiospheres within microcapsules facilitates controlled electrical stimulation of cardiosphere suspension cultures, for example, to mature engineered cardiac tissue constructs in vitro while still allowing for safe and facile (downstream) handling of mass-produced cardiospheres^103–105^.

Microencapsulated cardiospheres contractility was quantified using hPSCs that were genetically modified to express fluorescent α-Actinin/mRubyII fusion protein to visualize the cell’s sarcomere Z-lines, which allowed for time-resolved analysis in live cells (Fig. 5n, Supplementary Fig. S16, Supplementary Video 4)^106,107^. Specifically, we measured the shortening of sarcomeres on a single-cell level by determining the length between two adjacent Z-lines during contraction events. This revealed that sarcomeres contracted to 82 ± 9% of their original length upon 5 V external pacing (1.68 ± 0.17 µm in original state and 1.43 ± 0.17 µm in contraction) (Fig. 5o–q), which is in line with findings in literature (1.73 ± 0.015 μm in original state and 1.54 ± 0.014 μm in contraction, which correlates to 89% of its original length)^107^. In addition, contraction was also quantified in terms of fraction area change, which revealed a 0.024 ± 0.016 fraction area change upon contraction (Fig. 5r), which was not readily quantifiable using fraction area change within conventional cardiac cultures (Supplementary Video 5), thus offering improved functional read-outs for various applications such as developmental biology and drug testing.

To conclude, we report on a microfluidic production platform that can produce cellular spheroids in a clean manner (i.e., oil-free and surfactant-free) at a rate that is orders of magnitude faster than conventional approaches, with high control and resolution, which uniquely enables the production of these living 3D microtissues at clinically and industrially relevant scales. Specifically, a triple-jet strategy was designed to controllably collide microdroplets of distinct chemical compositions in-air, in an in-line fashion, to produce hollow thin-shelled hydrogel microcapsules, which were demonstrated to be capable of acting as highly efficient microtissue-forming bioreactors. These mass-produced microcapsules allowed hPSCs to autonomously undergo lumenogenesis to form cavitated pluripotent spheroids with polarized cytoarchitecture. Exposing mass-produced hESC-laden microcapsules to myocardial differentiation medium resulted in the production of high-quality functional cardiospheres that were composed of a homogenous cell population, as confirmed by single-cell RNAseq. Taken together, we here present a microfluidic platform for the mass production of 3D functional living microtissues which opens new areas of research and drives clinical and industrial adoption in application areas such as tissue engineering, drug testing, and cultured meat.

## Methods

### Triple-jet in-air microfluidic setup

The IAMF setup for hollow microgel formation consisted of three tapered nozzles with various diameters of which the second and third nozzle were placed under ±40° on the left and right of the central nozzle respectively, and were aligned using micrometer-precision XYZ-stages. The nozzles were connected with fluorinated ethylene propylene tubing (FEP) to Luer-lock glass syringes, of which the flow rate was controlled by Cetoni neMESYS low-pressure syringe pumps. The central nozzle was vibrated using a piezoelectric actuator, which was operated at 5 Vpp and frequencies between 1 and 10 kHz, allowing controlled breakup of the microjet. Depending on the nozzle diameter used, an optimized piezoelectric frequency was used to achieve a monodisperse breakup of the microjet in microdroplets. The microjet of the second nozzle was aligned such that it coalesced with the formed droplet train in a stable manner, which was repeated for the microjet of the third nozzle. Proper droplet breakup and coalescence of the microjets were confirmed using a high-speed microscope camera (IDS, UI-3240CP Rev. 2).

### Alginate hollow microcapsule production and analysis

The production method of alginate hollow microcapsules was, unless stated otherwise, achieved as follows. The first microjet consisted of 10% (w/v) dextran and 50 mM calcium chloride (CaCl_2_) in dH_2_O, the second microjet consisted of 0.2% (w/v) alginate and 10% (v/v) ethanol (EtOH) in H_2_O, and the third microjet consisted of 0.2 M CaCl_2_ and 55% (v/v) EtOH in dH_2_O. By using different nozzle diameters, it was possible to produce alginate hollow microcapsules in 3 different size regimes; being small (180 ± 8 µm), medium (243 ± 9 µm), or large (459 ± 17 µm). For small microcapsules, nozzle diameters of 50 µm, 50 µm, and 100 µm, with flow rates of 0.9 ml/min, 1.1 ml/min, and 1.5 ml/min were used respectively for the first, second, and third microjet with an actuator frequency of 5.5 kHz. For medium size microcapsules, nozzle diameters of 100 µm, 100 µm, and 150 µm with flow rates of 2 ml/min, 2.2 ml/min, and 2.6 ml/min were used respectively for the first, second, and third microjet with an actuator frequency of 5.5 kHz. For large-size microcapsules, nozzle diameters of 150 µm, 150 µm, and 250 µm with flow rates of 3 ml/min, 3.2 ml/min, and 4.6 ml/min were used respectively for the first, second, and third microjet with an actuator frequency of 2.5 kHz. Produced hollow microcapsules were collected in a 0.1 M CaCl_2_ in a dH_2_O collection bath. The microjets were characterized by We ± 25. Using collisional angles of ±40°, this corresponded to We_impact_ ± 10. Produced hollow microcapsules were visualized using brightfield microscopy (EVOS FL Imaging System, Thermo Fisher). Size distribution, monodispersity, circularity, amount of cores and shell thickness were analyzed using ImageJ software. To visualize the hollow nature of the microcapsules, 0.5 mg/ml 2000 kDa Dextran-FITC was added to the second microjet when producing the hollow microcapsules in order to achieve a fluorescent shell of the hollow microcapsules. Fluorescently labeled hollow microcapsules were then analyzed by confocal z-stack microscopy analysis (Nikon A1 confocal laser microscope). Permeability experiments were performed by 30 min incubation of microcapsules in a bath containing 0.5 mg/ml Dextran-FITC of 150 kDa, 500 kDa, and 2000 kDa before visualization using fluorescent confocal microscopy (Nikon A1 confocal laser microscope).

### PEGDA hollow microcapsule production

Poly(ethylene glycol) diacrylate (PEGDA) hollow microcapsules were produced in an identical manner as small alginate microcapsules, with the exceptions that 10% (v/v) PEGDA and 0.25% (w/v) and Irgacure 2959 were additionally added to the second microjet. The third microjet was aligned such that the impact took place 8.9 ms after the second microjet coalesced with the first microjet, which corresponded to a height difference of 8 cm between the second and third microjets. A UV lamp (LAMP) was aimed at the collection bath and was used to photocrosslink the PEGDA in the shell of the alginate hollow microcapsules during production and for 1 min after collection at 25% UV power. After this, a 1% EDTA in dH_2_O wash was used to remove the alginate. The resulting PEGDA hollow microcapsules were analyzed using brightfield microscopy.

### Dex-TA hollow microcapsule production and analysis

Tyramine-conjugated dextran (Dex-TA) was synthesized as described previously^108^. In short, dextran was activated with a *p*-nitrophenyl carbonate group (PNC) and dissolved in DMF, after which TA was added under nitrogen. The degree of substitution of the produced Dex-TA was determined to be 15 by proton-NMR. Dex-TA hollow microcapsules were produced in an identical manner as small alginate microcapsules, with the exception that 5% (w/v) Dex-TA and 57.5 U/ml horseradish peroxidase (HRP) were additionally added to the second microjet. The collection bath additionally contained 0.1% H_2_O_2_ in H_2_O to allow for enzymatic crosslinking. After 1 min of the enzymatic crosslinking post-cure, the hollow microcapsules were washed with 1% EDTA to remove the alginate. The resulting Dex-TA hollow microcapsules were stained with 30 µM of ethidium homodimer (EthD-1) and analyzed using brightfield microscopy and fluorescence confocal microscopy.

### Oil residue in chip-based approaches

Chip-based hollow microcapsule production was performed as described previously^54,73^. In short, a polymethyl methacrylate (PMMA) microfluidic chip was used in a flow-focusing setup to produce droplets consisting of 5% Dex-TA and 250 U/ml HRP within an oil phase of n-hexadecane with the addition of 1% (v/v) Oil-red-O and 1% span 80 as a surfactant. The resulting emulsion was broken by three consecutive washes using n-hexadecane without surfactant and a subsequent wash with phosphate-buffered saline (PBS). Absorption of n-hexadecane with and without Oil-red-O and hollow microcapsules produced using chip-based microfluidics or IAMF was measured using spectrophotometry (Multiskan Go Microplate; Thermo Scientific) at 512 nm.

### Cell culture

3T3 Fibroblasts (ATCC, CRL-1658) were cultured in Dulbeco’s Modified Eagle Medium (DMEM) supplemented with 10% Fetal Bovine Serum (FBS), 50 µM 2-mercapto-ethanol, 100 U/ml penicillin and 100 µg/ml streptomycin. The culture medium was changed biweekly when 80% confluency was reached. Cells were kept in a humidified environment at 37 °C with 5% CO_2_ before use.

The dual fluorescent human pluripotent stem cells (human embryonic stem cells) reporter lines MESP1(mCherry)/NKX2-5(eGFP)^101^ derived from hESC HES3 (female) (ESIBI), hPSCreg database: ESIBIe003-A clone 3A1 hetero zygote, and ACTN2(mRubyII)/NKX2-5(eGFP)^106^ derived from hESC HES3 (female) (ESIBI), hPSCreg database: ESIBIe003-A clone 3A1 hetero zygote. were cultured in Essential 8 medium (E8) (Thermo Fisher) supplemented with 50 U/ml penicillin and 50 µg/ml streptomycin (Thermo Fisher) on Vitronectin (Thermo Fisher) coated plates. Cells were passaged twice a week and refreshed the day after passaging. Cells were cultured in a humidified environment at 37 °C with 5% CO_2_.

### Cell encapsulation

To optimize the cell loading concentration, 3T3 cells were fixed using a 10% buffered formalin solution (Thermo Fisher) and added to the first microjet solution in concentrations ranging from 10^6^ to 10^7^ cells/ml. The number of cells per gel was confirmed based on confocal microscopy analysis following EthD-1 staining of the fixed cells. For the production of live cell-laden alginate hollow microcapsules, dH_2_O in all microjet solutions was exchanged with DMEM without phosphates. Detached cells were washed with medium, flown through a 40 µm cell strainer, and suspended in the first microjet solution at a concentration of 10^7^ cells/ml. The cell-laden first microjet solution was loaded into an ice-cooled gastight syringe. Cell sedimentation and subsequent clumping in the syringe were prevented through the dextran crowder, the addition of a frequently stirred magnet, and by keeping the syringe on ice. Nozzle diameter and flow rate parameters were selected for medium-sized microcapsule production. Cell-laden hollow microcapsules were collected in a large collection bath containing 50 ml of 0.1 M CaCl_2_ in DMEM without phosphates in order to immediately dilute the EtOH to cell-viable concentrations below 2% (v/v). Cell-laden microcapsules were then immediately washed using a 70 µm cell strainer and resuspended in cell culture medium within 2 min upon collection. The cell-laden hollow microcapsules were transferred to culture plates for cell culture. For additional analyses, cell-laden hollow microcapsules were first washed with PBS and fixated using a 10% formalin solution. Cells were permeabilized using 0.1% Triton X-100 and subsequently stained with 2.5 U/ml of phalloidin-AF488 and 10 of µg/ml DAPI to stain F-actin and nuclei respectively and analyzed by confocal microscopy (Nikon confocal A1). Cell viability of cell-laden hollow microcapsules was studied using calcein AM and ethidium homodimer-1 staining according to the manufacturer’s protocol (Invitrogen) and imaging using a digital fluorescence microscope (EVOS FL Imaging System, Thermo Fisher) in multiple independent experiments. Spheroids were retrieved from their microcapsules via exposure to 10 U/ml of alginate lyase at day 0-4 or exposure to PBS after day 4.

### Pluripotent spheroid production

Human pluripotent stem cells (human embryonic stem cells, derived from hESC HES3 (female), hPSCreg database: ESIBIe003-A. Sequencing, karyotyping and marker analysis were performed for identification) were encapsulated using the In-Air cell encapsulation method as described above, using a concentration of 2*10^6^ cells/ml such that approximately 20 cells were encapsulated per microcapsule. During the first 24 h, cell-laden microcapsules were cultured in Essential 8 (E8) medium^109^ supplemented with PVA and ROCK inhibitor. Afterward, cell-laden microcapsules were cultured in E8 medium, which was refreshed every other day. Encapsulated embryoid bodies were analyzed for aggregation efficiency, cavitation efficiency, embryoid body diameter, and cavity diameter by brightfield microscopy. Cell viability analysis was performed as described above. For microwell-mediated production of embryoid bodies, round-bottom 96-well plates were used for the production of 5000 cell aggregates, and alginate microwells with a diameter of 200 µm per well were used to produce 500 and 20 cell aggregates, as described by Moreira Teixeira et al.^110^. Cavitation was assessed via nuclei staining using DAPI as described above. The distance of nuclei to the hPSC spheroid centroid was measured in fluorescent confocal z-stacks of DAPI-stained embryoid bodies using ImageJ software. To analyze cellular orientation as well as nuclear position within the cell, live cells were exposed to Draq5 (5 µM) to stain nuclei, and CellMask Green (1000X dilution of stock) to stain cell membranes, which were analyzed using fluorescence confocal microscopy. To analyze pluripotency, cells were stained for Sox2 and Oct3/4 using antibodies (Sox2: eBioscience, 53-9811-82, 1:100 dilution. Oct3/4: Santa Cruz Biotechnology, sc-5279 AF647, 1:100 dilution). In short, embryoid bodies that were cultured for 6 days were permeabilized with 0.1% Triton in 0.5% BSA, blocked with 5% FBS, and stained with Sox2 and Oct3/4 antibodies at 1:100 and DAPI at 1:4000 in 1% BSA overnight. Stained embryoid bodies were analyzed using fluorescence confocal microscopy.

### Cardiosphere production

The dual fluorescent hPSC reporter lines MESP1(mCherry)/NKX2-5(eGFP) and ACTN2(mRubyII)/NKX2-5(eGFP) (human embryonic stem cells, derived from hESC HES3 (female), hPSCreg database: ESIBIe003-A. Sequencing, karyotyping and marker analysis was performed for identification) were encapsulated using the In-Air encapsulation method as described above, using a concentration of 2*10^6^ cells/ml. During the first 24 h, cultures were exposed to E8 medium supplemented with PVA and Y-27632 ROCK inhibitor. From day 1 till day 4 cultures were exposed to BPEL medium^111^ containing, 30 ng/ml Activin A, 30 ng/ml BMP-4, 1.5 µM CHIR99021, 30 ng/ml VEGF, and 40 ng/ml SCF (Stem Cell Factor) in order to induce mesoderm differentiation^112^. The medium was refreshed at the end of day 2. From day 4 till day 22 cultures were exposed to BPEL medium without supplements, which was refreshed 2/3 times per week. Activation of the *MESP1* and *NKX2-5* genes were analyzed for encapsulated cardiospheres using fluorescence confocal microscopy (mCherry and eGFP, respectively). The percentage of NKX2-5/eGFP positive cells was determined by FACS analysis after trypsinization of cardiospheres, which were cultured for 22 days. Calcium flux of encapsulated cardiospheres was analyzed using Fluo-4 AM (Thermo Fisher) calcium imaging (5 µM) measuring fluorescence intensity over time using fluorescence microscopy. To electrically stimulate encapsulated cardiospheres, two platinum electrodes (Advent Research Materials) connected to a custom-made pacing device were placed in a cardiosphere containing well of a 96-wells plate and paced with 5 V (10 ms biphasic pulses, 7–10 V/cm²) at frequencies ranging from 0.5 to 2 Hz. α-Actinin (mRubyll) was imaged using fluorescence microscopy (Nikon ECLIPSE Ti2 inverted microscope). Both sarcomere contraction and contraction amplitude were analyzed by means of fraction area change using ImageJ software.

### Single-cell RNA sequencing

Encapsulated EBs and cardiospheres were harvested at day 6 and day 21 respectively by dissolving the microcapsule using alginate lyase as described in the cell encapsulation section. Cells were dissociated into single-cell suspensions using TrypLE (Gibco) and sorted into 384-well plates containing primers with unique molecular identifiers (UMI), unique cellular barcodes and an oligo-(dT-N) for tagging the mRNA molecules. Single-cell RNA sequencing barcode file is added as a supplementary data file in Supplementary Dataset 1. Directly after sorting, the plates were spun down and stored at −80 °C until library preparation. The sorted 384-well plates were processed using a modified CEL-Seq2 protocol^113^. In short, ERCC spike-in (1:50,000) was dispensed using a Nanodrop II (BioNex Solutions Inc.) in each well. After a 5 min incubation step at 65 °C, 150 nl of the Reverse Transcription (RT) mix was dispensed into each well, and the first strand synthesis was performed with the following thermal cycles: 4 °C 5 min, 25 °C 10 min, 42 °C 1 h, 70 °C 10 min. Within an incubation of 2 h at 16 °C the second strand synthesis with *E. coli* DNA polymerase and *E. coli* DNA ligase (New England Biolabs), supplemented with *E. coli* RNase H (Invitrogen), was performed in the plate. After each of the dispensing steps, the plates were spun down at 1000 × *g* for 1 min at 4 °C. Subsequently, the cDNA from the wells were pooled per plate and purified using AmpureXP (Beckman Coulter). Overnight in vitro transcription (Ambion MEGAScript; Invitrogen) for 14 h at 37 °C, was followed by a 20 min at 37 °C exonuclease step and chemical fragmentation of the amplified RNA. The sample was purified using the AmpureXP beads, after which a library RT and amplification PCR (with 9 cycles) was performed to introduce unique sample indices to the plate libraries. After a final beads purification, the sample was quantified using the Denovix HS dsDNA assay and checked for library quality using the BioAnalyzer (Agilent Technologies). The samples were sequenced on the NextSeq 500 (Illumina) platform, at a depth of 20 million reads per plate. Trimming of the reads and alignment was performed with seq2science v0.6.0 (available in Zenodo, 10.5281/zenodo.4451349). In short, kb-python v0.26.4^114^ was used (kb count with the setting ‘--technology 1,8,16:1,0,8:0,0,0’) for mapping against the GRCh38 genome and quantified the UMI counts per cell. The Scater package v1.20.1^114^ was used for pre-processing and quality control. Only cells with a minimum of 500 detected genes and 1000 total counts, and a maximum of 50% mitochondrial transcripts and 20% of ERCC transcripts were kept in the analysis. Genes with an expression of 1 count in at least 2 cells were kept. Normalization and downstream analysis, such as dimensionality reduction and Louvain clustering were performed with the Seurat package v4.0.4^115^. The SCTransform function was used to normalize the data and select the 2000 highly variable genes (HVG) in the dataset. The cells were clustered using a shared nearest neighbor (SNN) modularity optimization-based clustering algorithm with a resolution of 0.9 and were projected onto Uniform Manifold Approximation and Projection (UMAP) using the first 10 principle components (PCs). Differential gene expression analysis between conditions was performed with a *t*-test in the FindMarkers function of Seurat, on the genes expressed in at least 20% of the cells in either of the clusters. The resulting differential gene lists were filtered for a log-fold change >1 and a *p*-adjusted value of <0.05 and the ComplexHeatmap package^115^ was used to visualize the sets of genes. Gene Ontology enrichment analysis was performed with the clusterProfiler v4.0.5 package^116^.

### Statistics

Sample size per experiment is reported in figure descriptions. Significance was determined based on one-way ANOVA analysis. The significance of *p* < 0.05 is indicated by *. All statistical analyses were performed in OriginPro2017.

### Reporting summary

Further information on research design is available in the Nature Portfolio Reporting Summary linked to this article.

### Supplementary information

Supplementary Information

Description of Additional Supplementary Files Document

Supplementary Dataset 1

Supplementary Movie 1

Supplementary Movie 2

Supplementary Movie 3

Supplementary Movie 4

Supplementary Movie 5

Reporting Summary

### Source data

Source Data

## Data Availability

The single-cell RNA sequencing data generated in this study have been deposited in the Gene Expression Omnibus database under accession number “GSE219249”. Single-cell RNA sequencing barcode file is added as a Supplementary Dataset 1. All other data supporting the findings of this study are available within the article and the Source Data file. Any additional requests for information can be directed to, and will be fulfilled by, the lead contact. Source data are provided with this paper.
