# Supplementary material for: Mass production of lumenogenic human embryoid bodies and functional cardiospheres using in-air-generated microcapsules
Source: Nat Commun. 2023 Oct 21;14:6685. doi: 10.1038/s41467-023-42297-0 (PMC10590445; doi:10.1038/s41467-023-42297-0)
Supplement: Supplementary file 1 — Supplementary Information [file 41467_2023_42297_MOESM1_ESM.pdf]

**Supporting Information**

# Mass Production of Lumenogenic Human Embryoid Bodies and Functional Cardiospheres using In-Air Generated Thin-shelled Microcapsules

Bas van Loo[1], Simone A. ten Den[2], Nuno Araújo-Gomes[1], Vincent de Jong[1], Rebecca R. Snabel[3], Maik Schot[1], José M. Rivera-Arbeláez[2,4], Gert Jan C. Veenstra[3], Robert Passier[2,5], Tom Kamperman[1,6], and Jeroen Leijten[1]

[1] University of Twente, TechMed Centre, Department of Developmental BioEngineering, Enschede, The Netherlands

[2] University of Twente, TechMed Centre, Department of Applied Stem Cell Technology, Enschede, The Netherlands

[3] Radboud University, Radboud Institute for Molecular Life Sciences, Faculty of Science, Department of Molecular Developmental Biology, Nijmegen, The Netherlands

[4] University of Twente, TechMed Centre, Max Planck Center for Complex Fluid Dynamics, BIOS Lab-on-a-Chip Group , Enschede, The Netherlands

[5] Leiden University Medical Centre, Department of Anatomy and Embryology, Leiden, Netherlands

[6] IamFluidics B.V., De Veldmaat 17, 7522NM Enschede, The Netherlands.

## Supplemental figures

**Supplemental figures**

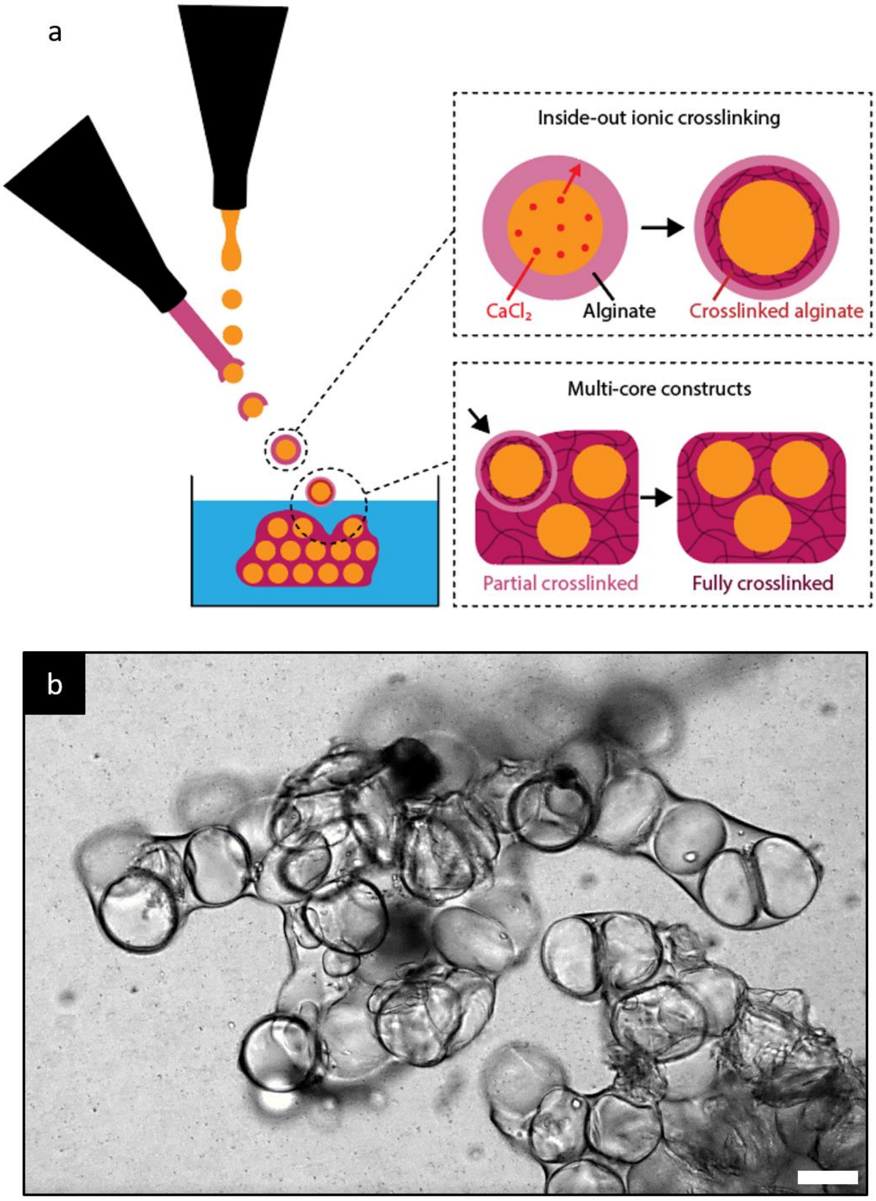


**Figure S1. In Air microfluidics using a two-nozzle setup results in formation of uncontrolled multi-compartment microparticles.** (a) Schematic of two-nozzle setup, inside-out crosslinking process, and mechanism by which multi-compartment microparticles are formed. (b) Microphotograph of formed microparticle using two-nozzle in-air microfluidics. Scale bar represents 200 µm.

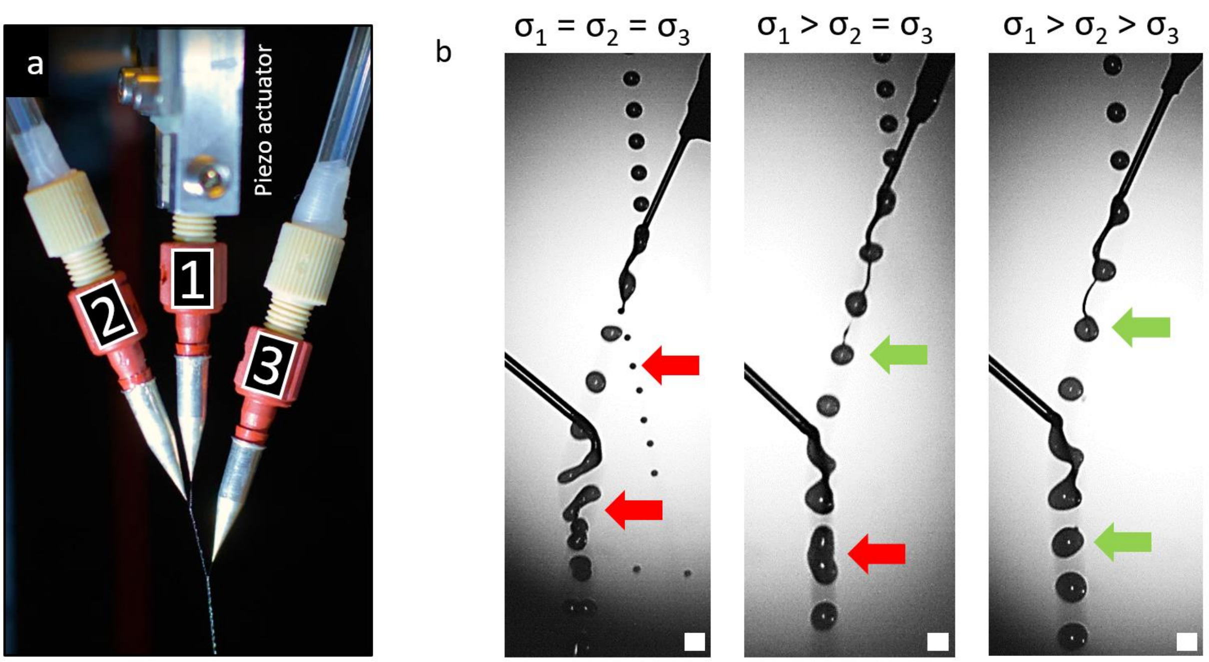


**Figure S2. In Air microfluidics three-nozzle setup for In Air droplet encapsulation.** (a) Photograph of a three nozzle In Air microfluidics setup, with a piezo-electric actuator attached to the first nozzle. (b) Microphotographs of the encapsulation process with $\sigma_1 = \sigma_2 = \sigma_3$, $\sigma_1 > \sigma_2 = \sigma_3$ and $\sigma_1 > \sigma_2 > \sigma_3$. Red arrows indicate spraying and improper encapsulation, green arrows indicate proper encapsulation. Scale bar represents 200 µm.

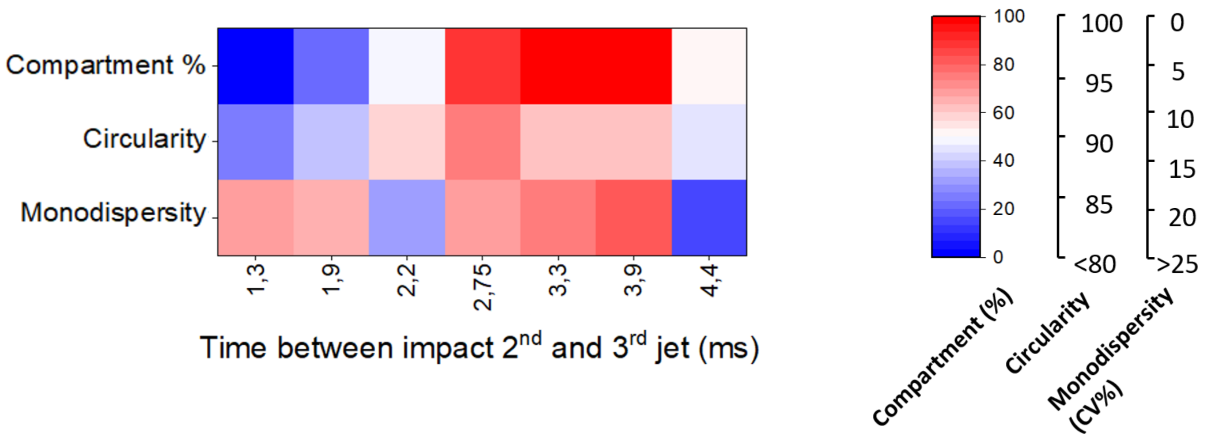


**Figure S3. Third jet coalescence location dictates hollow microcapsule formation.** Effect of in-air flight time before third jet coalescences with the droplet train as analyzed in terms of percentage of single compartment, circularity, and monodispersity of produced microparticles (n>85).

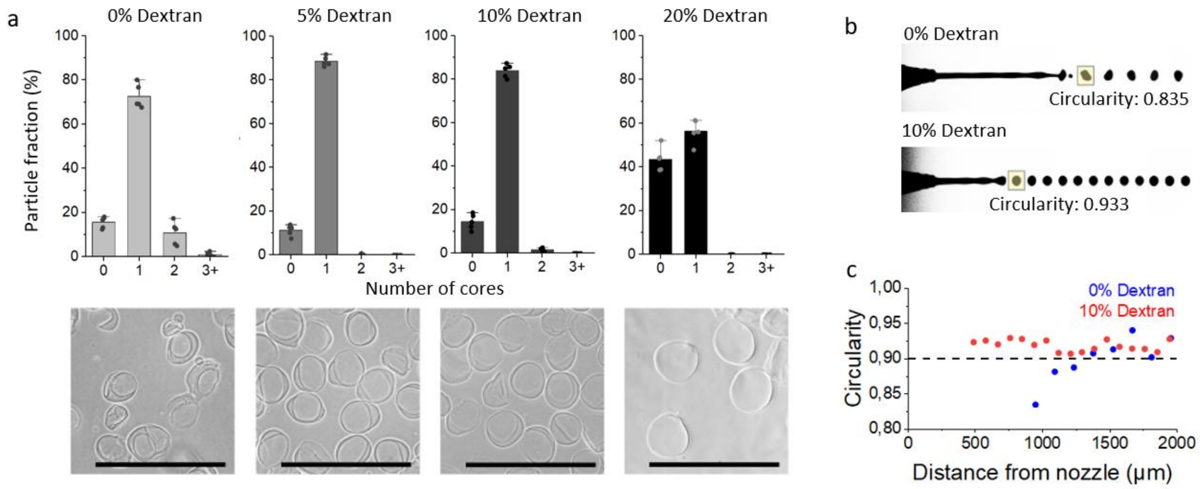


**Figure S4. Effect of dextran in core liquid on hollow microcapsule formation.** (a) Number of hollow compartments per microparticle using dextran core liquid concentrations of 0%, 5%, 10%, and 20%, including corresponding microphotographs (n=60). Data is presented as mean values +/- SD. (b) Microphotographs and (c) quantification of circularity of formed droplets with and without 10% dextran. Scale bar represents 400 µm.

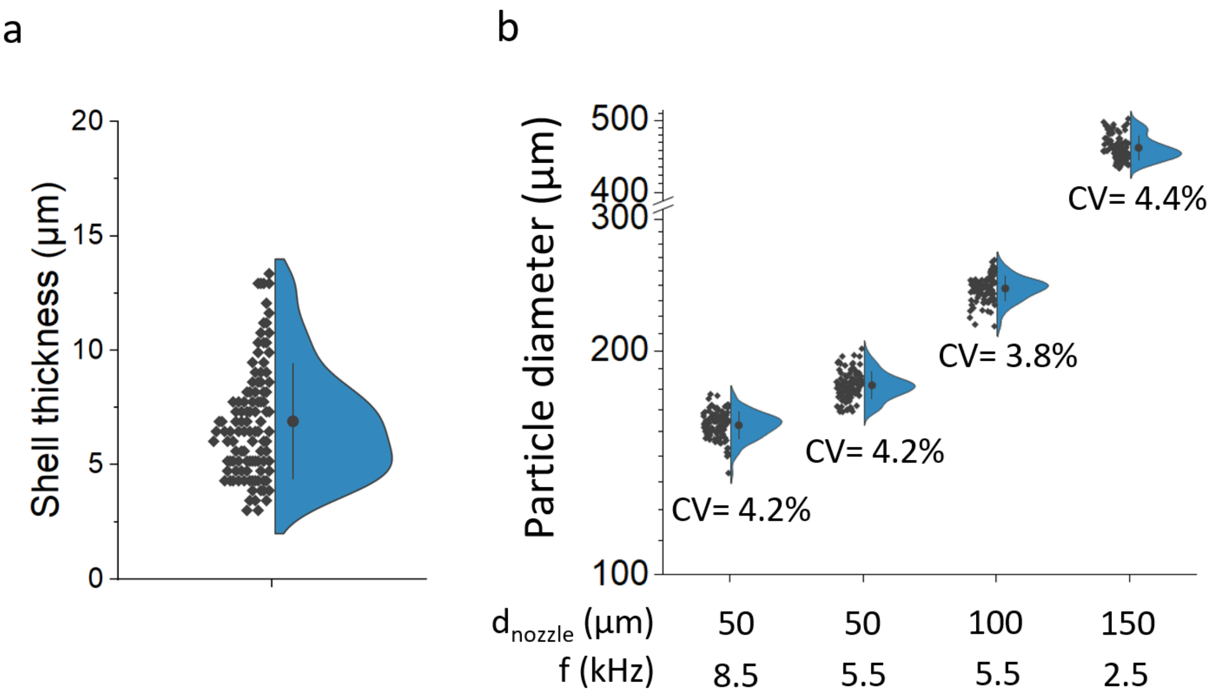

**Figure S5. Hollow microcapsule morphology.** (a) Quantification of shell thickness of in-air produced microcapsules (n=107). Data is presented as mean values +/- SD. (b) Quantification of microcapsule diameter and monodispersity using different nozzle sizes and different piezo-electric actuator frequencies (n=100). Data is presented as mean values +/- SD.

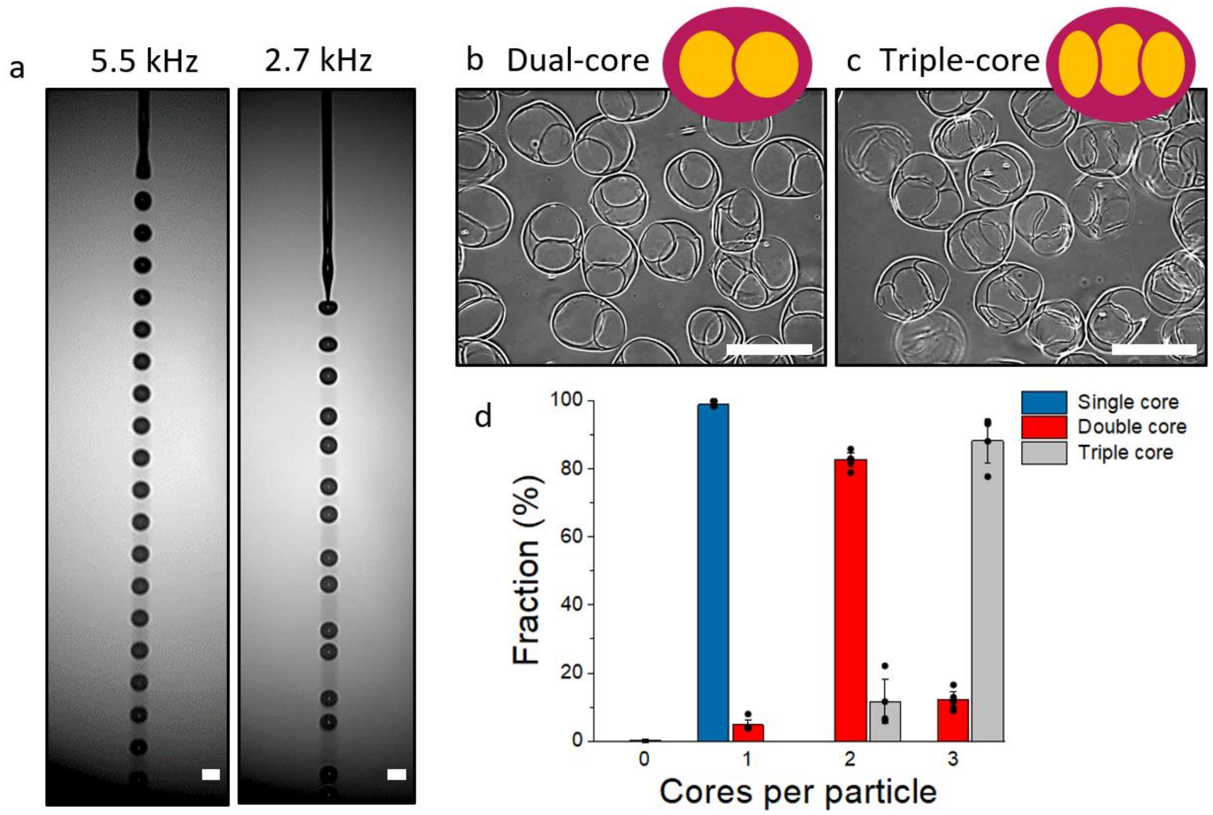


**Figure S6. Controlled production of multi-core microparticles by adjusting piezo-actuator frequency.** (a) Microphotographs of droplet formation at 5.5 kHz and 2.7 kHz. (b) Microphotograph of dual-core microcapsules (c) and triple-core microcapsules. (d) Quantification of percentage single-, dual-, and triple-core microcapsules (n=169 microparticles). Data is presented as mean values +/- SD. Scale bar represents 200 µm.

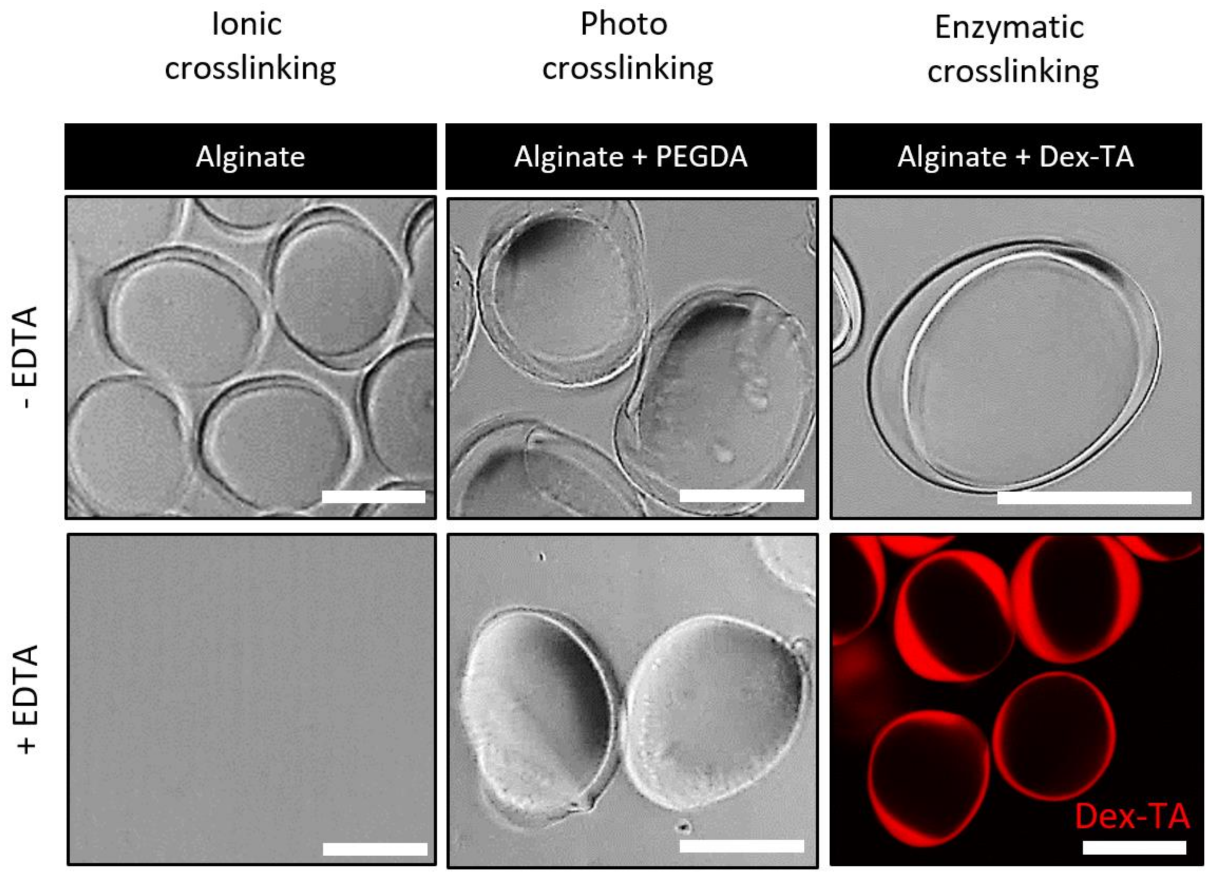


**Figure S7. In Air production of microcapsules using photocrosslinkable and enzymatic crosslinkable materials.** Microphotographs of ionically crosslinked alginate microcapsules, photocrosslinked PEGDA microcapsules, and enzymatic crosslinked dextran-tyramine microcapsules, before and after EDTA treatment. Dex-TA microcapsules post-EDTA were stained with ethidium homodimer (red). Scale bar represent 100 µm.

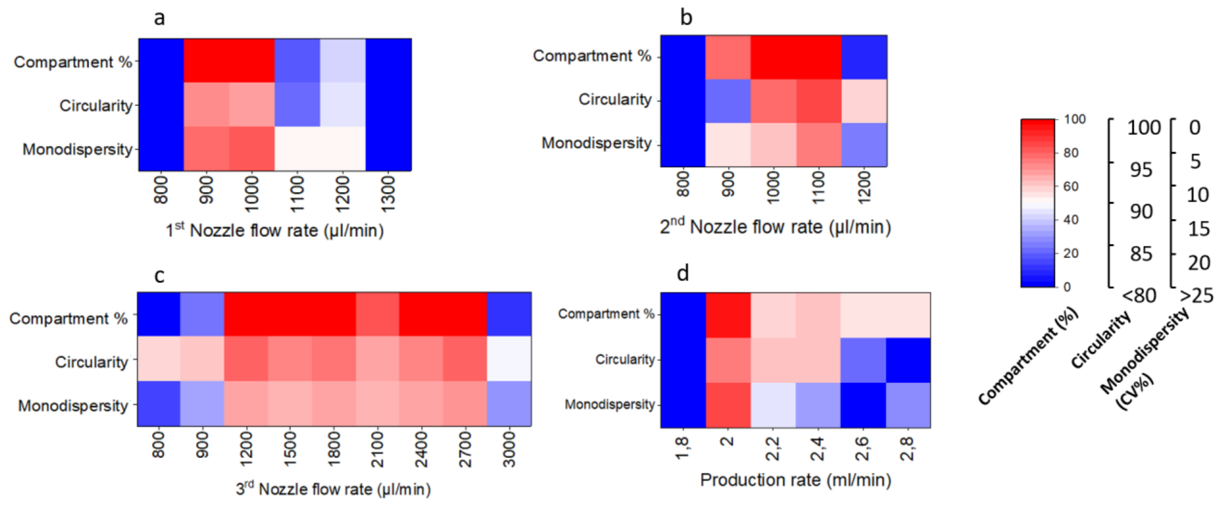


**Figure S8. Flow rate optimization for microcapsules production.** Quantification of the percentage of microparticles containing a single compartment, circularity of formed microparticles, and microparticle monodispersity. Setup used: Nozzle 1 (50 µm) at 900 µl/min, nozzle 2 (50 µm) at 1100 µl/min, nozzle 3 (100 µm) at 1500 µl/min. (a) Effect of first nozzle flow rate on microparticle formation (n=100). (b) Effect of second nozzle flow rate on microparticle formation (n=100). (c) Effect of third nozzle flow rate on microparticle formation (n=100). (d) Effect of total production flow rate on microparticle formation (n=100). Ratios between flow rates were kept constant at the ratio described above.

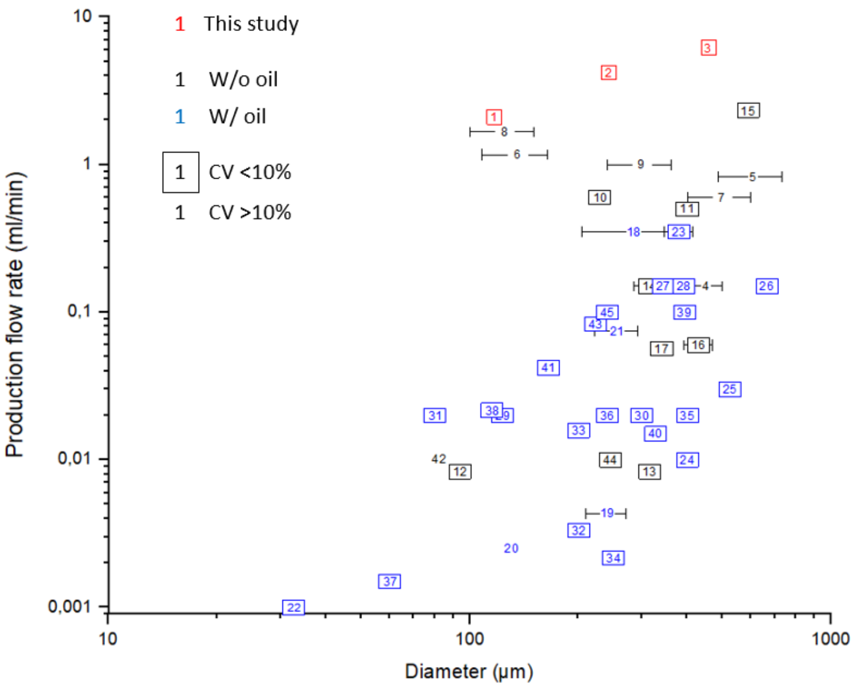


| | |
|---|---|
| 1-3 | This work |
| 4 | Aghdam et al.[86] |
| 5 | Correia et al.[44] |
| 6 | Alessandri et al.[46] |
| 7 | Ma et al.[47] |
| 8 | Alessandri et al.[45] |
| 9 | Rutkowski et al.[48] |
| 10 | Sakai et al.[48] |
| 11 | Ren et al.[69] |
| 12-13 | Song et al.[69] |
| 14 | Zhao et al.[79] |
| 15 | Domejean et al.[65] |
| 16 | Rao et al.[40] |
| 17-18 | Nguyen et al.[43] |
| 19 | Agarwal et al.[41] |
| 20 | Kim et al.[67] |
| 21 | Ashida et al.[64] |
| 22 | Seuss et al.[64] |
| 23 | Agarwal et al.[63] |
| 24 | Siltanen et al.[71] |
| 25 | Zhao et al.[78] |
| 26-28 | Watanabe et al.[75] |
| 29-31 | van Loo et al.[74] |
| 32 | Sun et al.[73] |
| 33 | Hu et al.[66] |
| 34 | Xie et al.[76] |
| 35 | Yang et al.[77] |
| 36 | Kamperman et al.[54] |
| 37 | Yu et al.[80] |
| 38 | Lin et al.[68] |
| 39 | Fattahi et al.[85] |
| 40 | Zhu et al.[84] |
| 41 | Jeong et al.[83] |
| 42 | Mytnyk et al.[49] |
| 43 | Wang et al.[81] |
| 44 | Liu et al.[39] |
| 45 | Liu et al.[82] |

**Figure S9. Literature study for continuously produced hollow core-shell microcapsules.**

Elaboration of figure 2l with corresponding studies per datapoint.

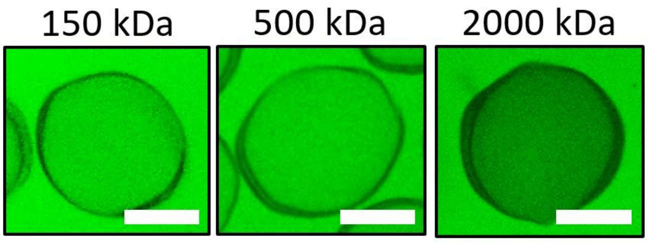


**Figure S10. Permeability of microcapsules.**

Fluorescent confocal micrographs of microcapsules incubated for 30 minutes in Dextran-FITC containing solutions of 150 kDa, 500 kDa and 2000 kDa. Representative micrographs out n=3 experiments. Scale bars represent 100 µm.

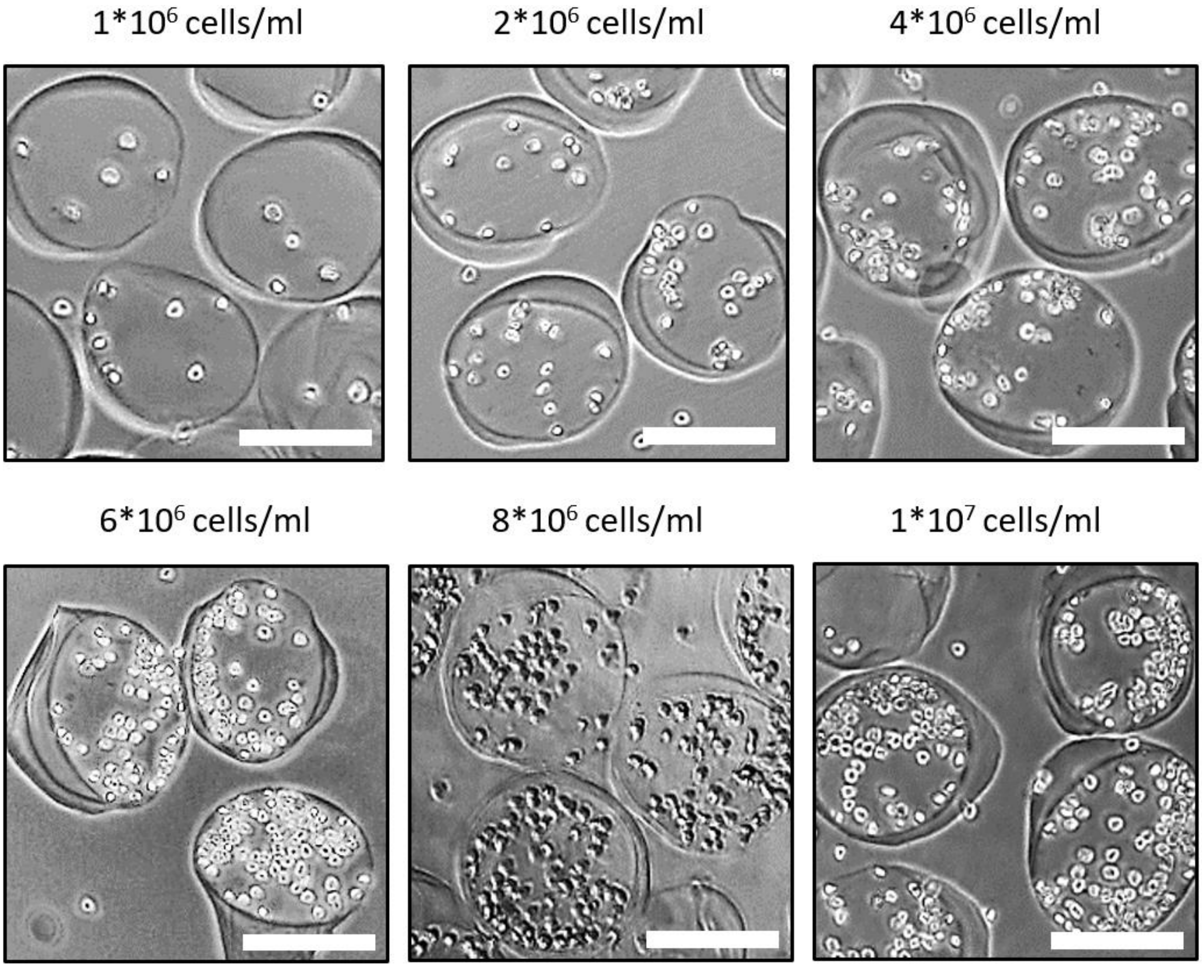


**Figure S11. In Air microencapsulation of 3T3 Fibroblast within alginate microcapsules.** Microphotographs of 3T3 fibroblast-laden microcapsules produced using a variety of cell concentrations. Representative micrographs out n=3 experiments. Scale bars represent 200 µm.

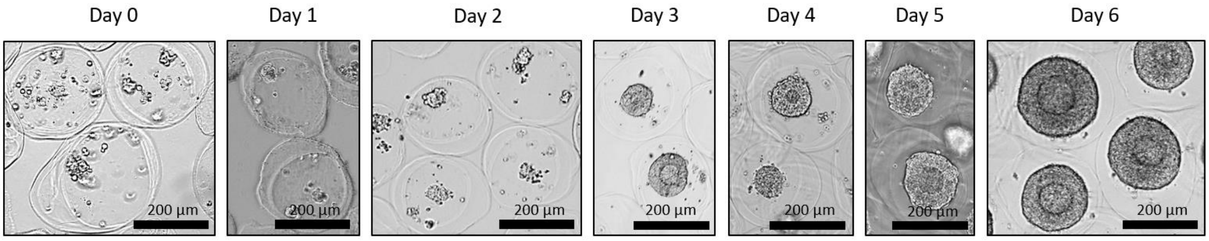


**Figure S12 Embryoid body formation by in-air microcapsulated hPSCs.** Microphotographs of encapsulated hPSCs show aggregation and lumenogenesis. Representative micrographs out n=3 experiments. Scale bar represents 200 µm.

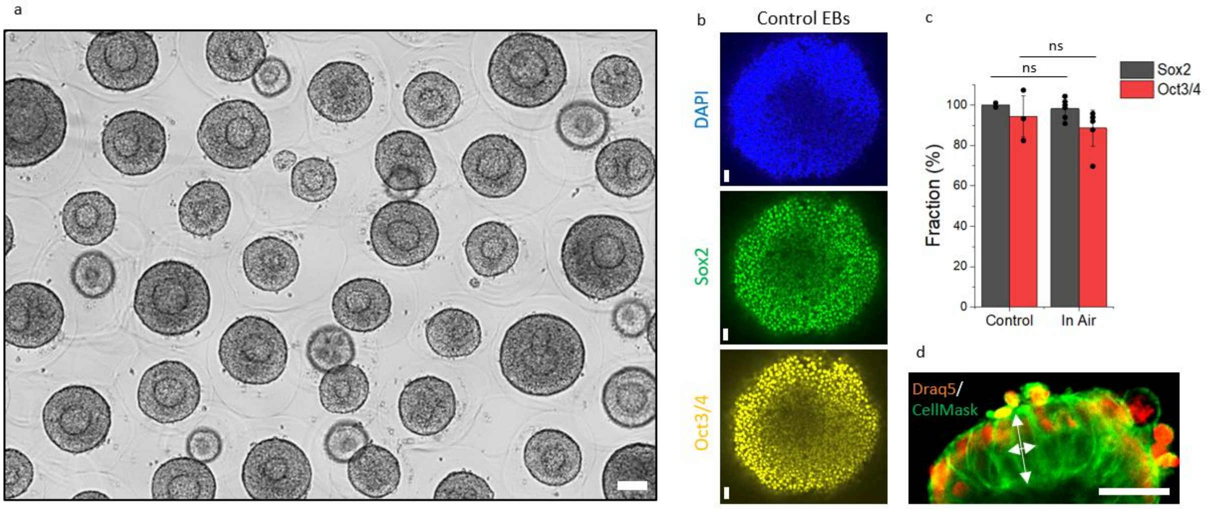


**Figure S13. Mass production of embryoid body by in-air microcapsulating hPSCs.** (a) Microphotograph of EBs formed within in-air produced alginate microcapsules. Representative micrographs out n=3 experiments. (b) Fluorescence microphotograph of EBs produced using microwells with 5000 cells/microwell, which were stained with DAPI for nuclei (blue), SOX2 (green), and OCT3/4 (yellow) for pluripotency. (c) Quantification and comparison of pluripotency by percentage of cells positive for SOX2 and OCT3/4 between EBs produced with conventional microwells or In Air microfluidics (n=300 cells). Data is presented as mean values +/- SD. Significance was determined based on one-way Anova analysis. Significance was determined based on one-way Anova analysis.

Significance of p <0.05 is indicated by *. (d) Confocal microphotograph of an EB with its nucleus stained with Draq5 (orange) and its cell membrane stained with CellMask (green). Scale bars represent 50 µm.

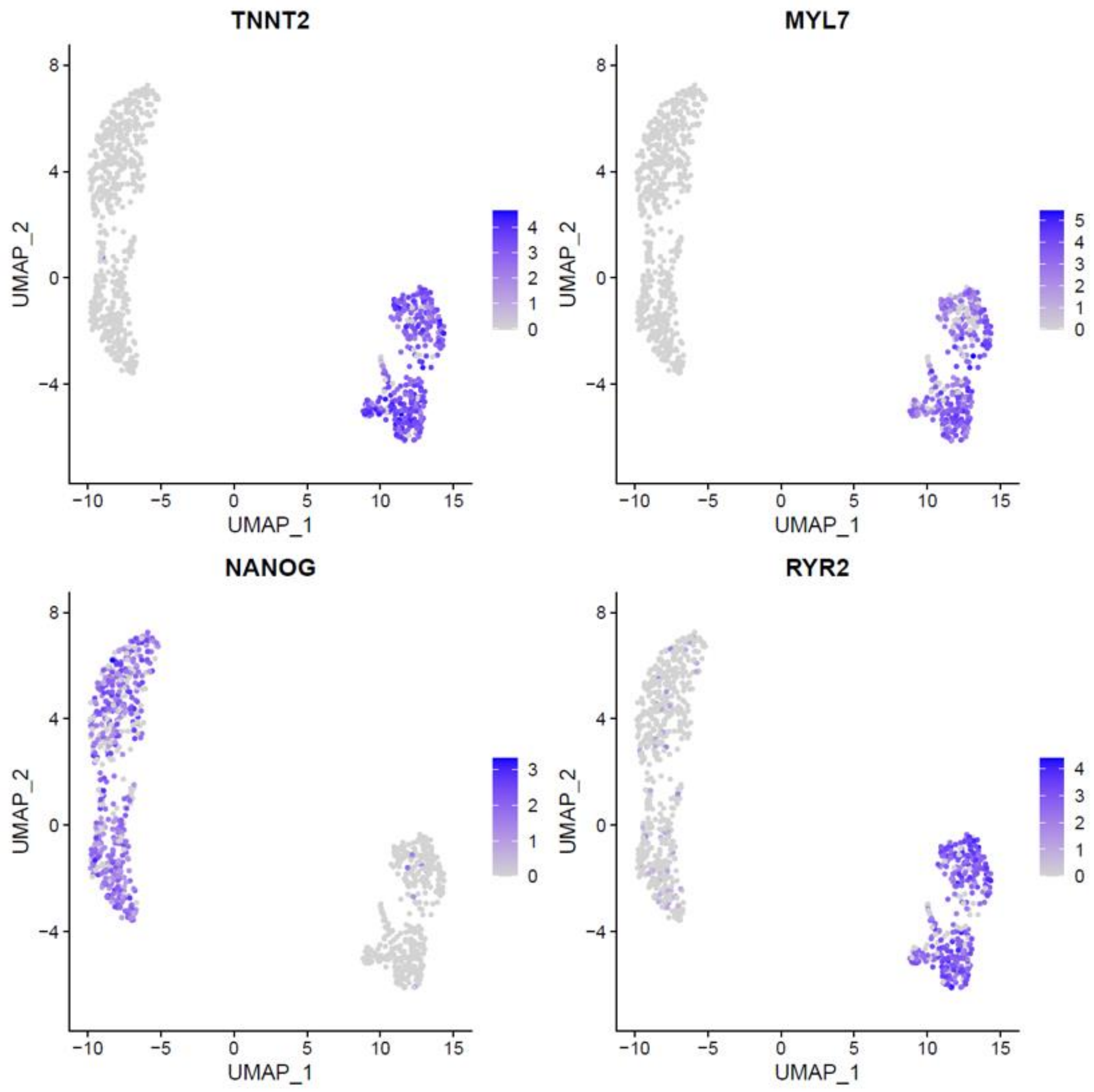


**Figure S14. Single cell RNA sequencing UMAP plots for pluripotency and cardiac markers.** Heatmap of NANOG, TNNT2, MYL7 and RYR2 gene expression in UMAP plots.

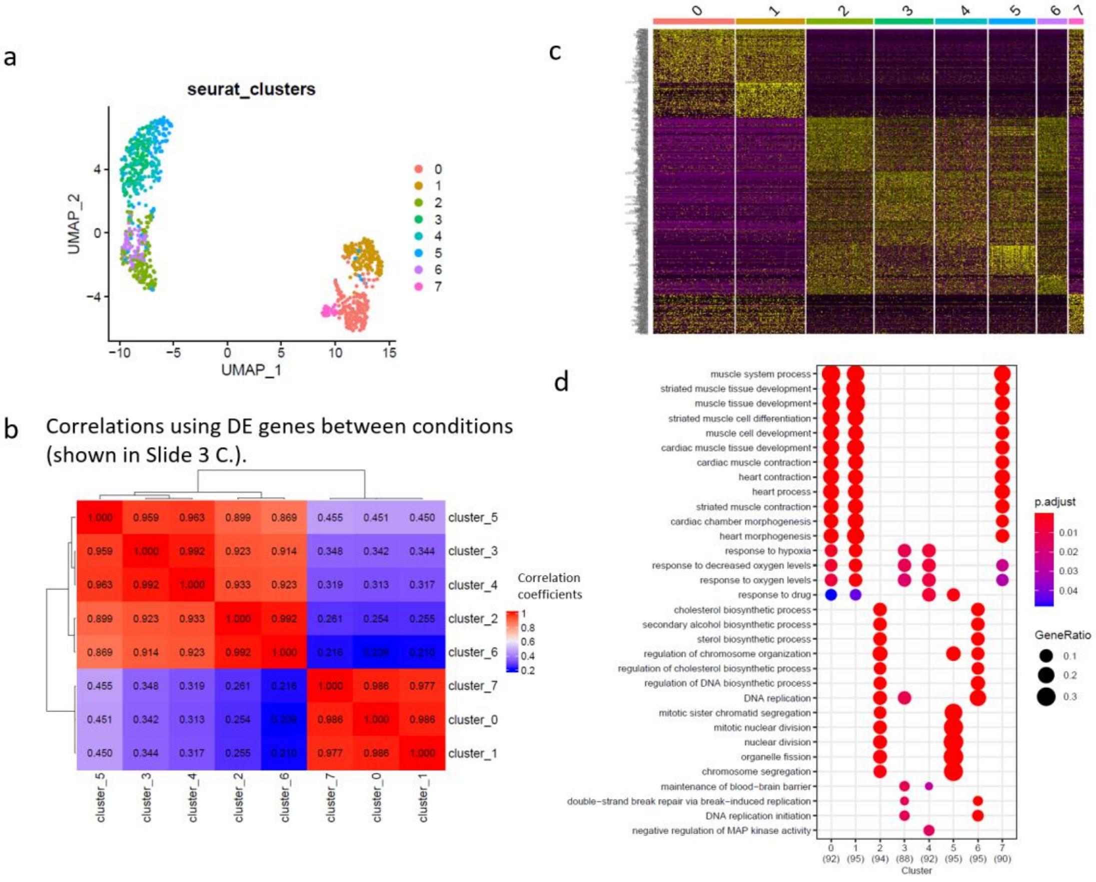


**Figure S15. Single cell RNA sequencing Seurat cluster data.** (a) Seurat clusters. (b) Correlation using differential expression genes between conditions. (c) Heatmap of marker genes corresponding to identified clusters. (d) Gene ontology heatmap of differential genes per cluster. P-adjust corrected using Benjamini-Hochberg correction for multiple comparisons.

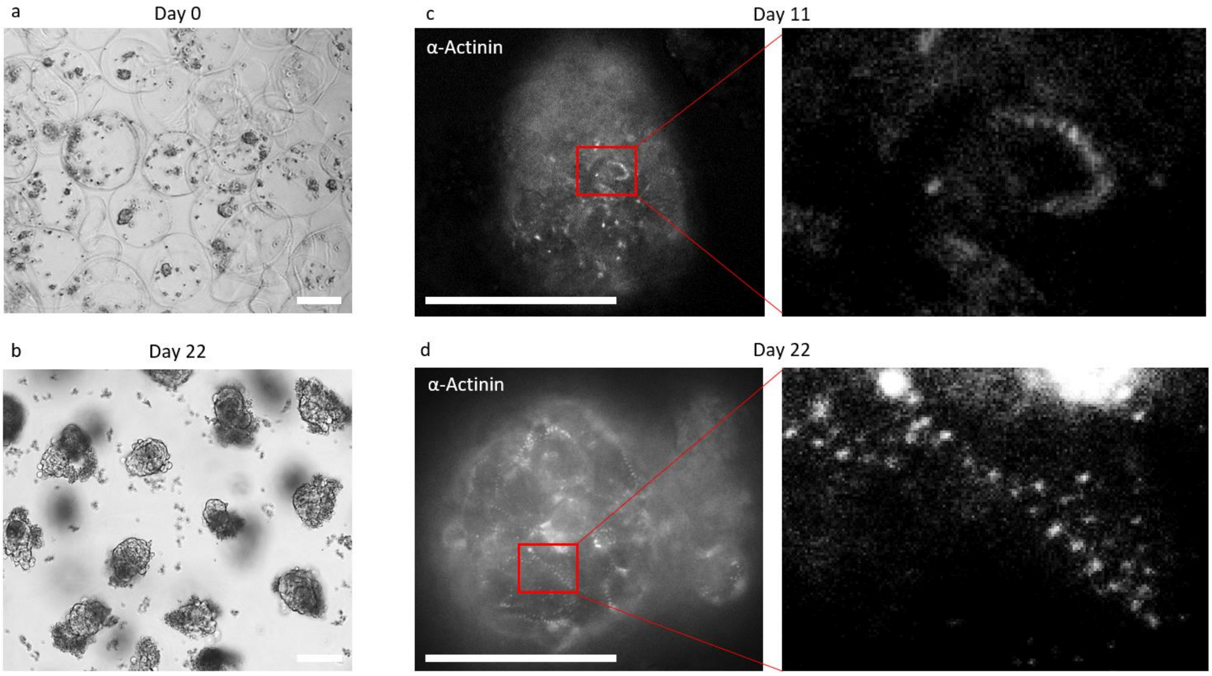


**Figure S16. Functional *human* cardiospheres produced in mass using in-air microfluidics.** (a) Microphotograph of encapsulated *human* pluripotent stem cells immediate after microencapsulation. (b) Microphotograph of encapsulated cardiospheres after 22 days of cardiac differentiation. Fluorescence microphotograph of α-actinin reporter in differentiated cardiomyocytes cultured after (c) 11 days and (d) 22 days of culture. Scale bars represent 100 µm.
