# Supplementary material for: Mass production of lumenogenic human embryoid bodies and functional cardiospheres using in-air-generated microcapsules
Source: Nat Commun. 2023 Oct 21;14:6685. doi: 10.1038/s41467-023-42297-0 (PMC10590445; doi:10.1038/s41467-023-42297-0)
Supplement: Supplementary file 2 — Description of Additional Supplementary Files Document [file 41467_2023_42297_MOESM2_ESM.pdf]

<u>Description of Additional Supplementary Files</u>

Supplementary Data 1. Single cell RNA sequencing barcode

Supplementary Video 1. In Air Microfluidic setup for mass production of microcapsules. Sequential start flow of core jet, actuation of piezo element at 5.5 kHz, start flow shellprecursor jet and start flow crosslinker jet.

Supplementary Video 2. Spontaneous contraction of cardiospheres. Brightfield microscopy video of spontaneously contracting cardiospheres within In Air generated microcapsules.

Supplementary Video 3. Calcium flux of (non-)stimulated cardiospheres. Calcium flux of cardiospheres within In Air generated microcapsules as visualized by Fluo-4 AM. Cardiospheres were stimulated at 0Hz, 0.5 Hz, 1 Hz and 2 Hz.

Supplementary Video 4. Sarcomere contraction in non-stimulated cardiospheres. Brightfield video, fluorescent microscopy video for NKX 2-5 GFP-positive and fluorescent microscopy video for alpha-actinin mCherry-positive sarcomeres in a cardiospheres within an In Air generated microcapsule.

Supplementary Video 5. Control plated cardiospheres. Brightfield video of control cardiospheres which were plated on a culture plate.
