# Supplementary material for: Mass production of lumenogenic human embryoid bodies and functional cardiospheres using in-air-generated microcapsules
Source: Nat Commun. 2023 Oct 21;14:6685. doi: 10.1038/s41467-023-42297-0 (PMC10590445; doi:10.1038/s41467-023-42297-0)
Supplement: Supplementary file 9 — Reporting Summary [file 41467_2023_42297_MOESM9_ESM.pdf]

nature portfolio

Corresponding author(s): Bas van Loo: srvanloo@gmail.com
Jeroen Leijten: Jeroen.leijten@utwente.nl

Last updated by author(s): Aug 10, 2023

Reporting Summary

Nature Portfolio wishes to improve the reproducibility of the work that we publish. This form provides structure for consistency and transparency in reporting. For further information on Nature Portfolio policies, see our Editorial Policies and the Editorial Policy Checklist.

Statistics

For all statistical analyses, confirm that the following items are present in the figure legend, table legend, main text, or Methods section.

n/a	Confirmed	
☐	☒	The exact sample size (*n*) for each experimental group/condition, given as a discrete number and unit of measurement
☐	☒	A statement on whether measurements were taken from distinct samples or whether the same sample was measured repeatedly
☐	☒	The statistical test(s) used AND whether they are one- or two-sided<br>*Only common tests should be described solely by name; describe more complex techniques in the Methods section.*
☐	☒	A description of all covariates tested
☐	☒	A description of any assumptions or corrections, such as tests of normality and adjustment for multiple comparisons
☐	☒	A full description of the statistical parameters including central tendency (e.g. means) or other basic estimates (e.g. regression coefficient) AND variation (e.g. standard deviation) or associated estimates of uncertainty (e.g. confidence intervals)
☐	☒	For null hypothesis testing, the test statistic (e.g. *F*, *t*, *r*) with confidence intervals, effect sizes, degrees of freedom and *P* value noted<br>*Give P values as exact values whenever suitable.*
☒	☐	For Bayesian analysis, information on the choice of priors and Markov chain Monte Carlo settings
☒	☐	For hierarchical and complex designs, identification of the appropriate level for tests and full reporting of outcomes
☒	☐	Estimates of effect sizes (e.g. Cohen's *d*, Pearson's *r*), indicating how they were calculated

Our web collection on statistics for biologists contains articles on many of the points above.

Software and code

Policy information about availability of computer code

Data collection	No software was used.
Data analysis	ImageJ2, OriginPro 2017, seq2science v0.6.0, kb-python v0.26.4, Scater package v1.20.1, Seurat package v4.0.4, clusterProfiler v4.0.5 package, ComplexHeatmap package v3.17

For manuscripts utilizing custom algorithms or software that are central to the research but not yet described in published literature, software must be made available to editors and reviewers. We strongly encourage code deposition in a community repository (e.g. GitHub). See the Nature Portfolio guidelines for submitting code & software for further information.

Data

Policy information about availability of data

All manuscripts must include a data availability statement. This statement should provide the following information, where applicable:

- Accession codes, unique identifiers, or web links for publicly available datasets
- A description of any restrictions on data availability
- For clinical datasets or third party data, please ensure that the statement adheres to our policy

The data generated in this study are provided in the Supplementary Information/Source Data file. The single cell RNA sequencing data generated in this study have been deposited in the Gene Expression Omnibus database [https://www.ncbi.nlm.nih.gov/geo/query/acc.cgi?acc=GSE219249]. Single cell RNA sequencing barcode file is added as a supplementary data file barcode_384.tab.

Human research participants

Policy information about studies involving human research participants and Sex and Gender in Research.

Reporting on sex and gender	Not applicable
Population characteristics	Not applicable
Recruitment	Not applicable
Ethics oversight	Not applicable

Note that full information on the approval of the study protocol must also be provided in the manuscript.

Field-specific reporting

Please select the one below that is the best fit for your research. If you are not sure, read the appropriate sections before making your selection.

☒ Life sciences ☐ Behavioural & social sciences ☐ Ecological, evolutionary & environmental sciences

For a reference copy of the document with all sections, see nature.com/documents/nr-reporting-summary-flat.pdf

Life sciences study design

All studies must disclose on these points even when the disclosure is negative.

Sample size	Sample size was determined based on similar studies in this field.
Data exclusions	No data were excluded.
Replication	Experiments were replicated at least 2 times to ensure replicability. All attempts of replication were successful.
Randomization	Cell batches of the same cell line were assigned randomly to experimental and control groups.
Blinding	Blinding was not possible as experimental conditions were evident from the image data.

Reporting for specific materials, systems and methods

We require information from authors about some types of materials, experimental systems and methods used in many studies. Here, indicate whether each material, system or method listed is relevant to your study. If you are not sure if a list item applies to your research, read the appropriate section before selecting a response.

Materials & experimental systems

n/a	Involved in the study	
☐	☒	Antibodies
☐	☒	Eukaryotic cell lines
☒	☐	Palaeontology and archaeology
☒	☐	Animals and other organisms
☒	☐	Clinical data
☒	☐	Dual use research of concern

Methods

n/a	Involved in the study	
☒	☐	ChIP-seq
☒	☐	Flow cytometry
☒	☐	MRI-based neuroimaging

Antibodies

Antibodies used	Sox2: eBioscience, 53-9811-82, 1:100 dilution<br>Oct3/4: Santa Cruz Biotechnology, sc-5279 AF647, 1:100 dilution
Validation	Antibodies are validated in the following study: doi.org/10.1007/s10544-021-00556-1

Eukaryotic cell lines

Policy information about cell lines and Sex and Gender in Research

Cell line source(s)	3T3 Fibroblasts (ATCC)<br>Dual fluorescent hPSC reporter line MESP1(mCherry/NKX2-5(eGFP) (human embryonic stem cells, derived from hESC HES3 (female), hPSCreg database: ESIBIe003-A.)<br>Dual fluorescent hPSC reporter line ACTN2(mRubyII)/NKX2-5(eGFP) (human embryonic stem cells, derived from hESC HES3 (female), hPSCreg database: ESIBIe003-A.)
Authentication	Sequencing, karyotyping and marker analysis was performed for authentication.
Mycoplasma contamination	All cell lines tested negative for mycoplasma contamination.
Commonly misidentified lines<br>(See ICLAC register)	No commonly misidentified lines were used in this study.
